# Supplementary material for: p57Kip2 imposes the reserve stem cell state of gastric chief cells
Source: Cell Stem Cell. 2022 May 5;29(5):826–839.e9. doi: 10.1016/j.stem.2022.04.001 (PMC9097776; doi:10.1016/j.stem.2022.04.001)
Supplement: Document S1. Figures S1–S5 [file mmc1.pdf]

Cell Stem Cell, Volume *29*

Supplemental Information

p57^{Kip2} imposes the reserve stem cell state of gastric chief cells

Ji-Hyun Lee, Somi Kim, Seungmin Han, Jimin Min, Brianna Caldwell, Aileen-Diane Bamford, Andreia Sofia Batista Rocha, JinYoung Park, Sieun Lee, Szu-Hsien Sam Wu, Heetak Lee, Juergen Fink, Sandra Pilat-Carotta, Jihoon Kim, Manon Josserand, Réka Szep-Bakonyi, Yohan An, Young Seok Ju, Anna Philpott, Benjamin D. Simons, Daniel E. Stange, Eunyoung Choi, Bon-Kyoung Koo, and Jong Kyoung Kim

Figure S1

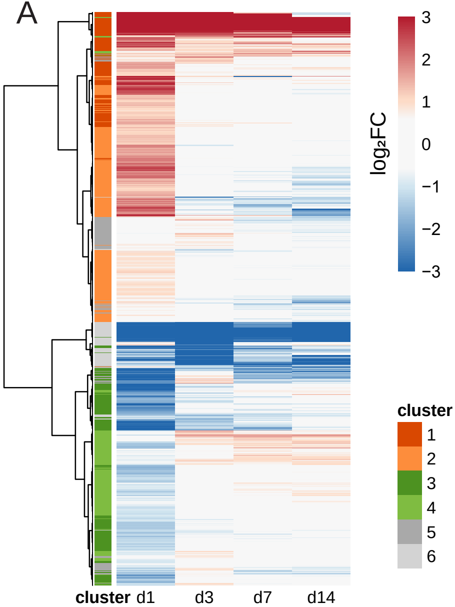

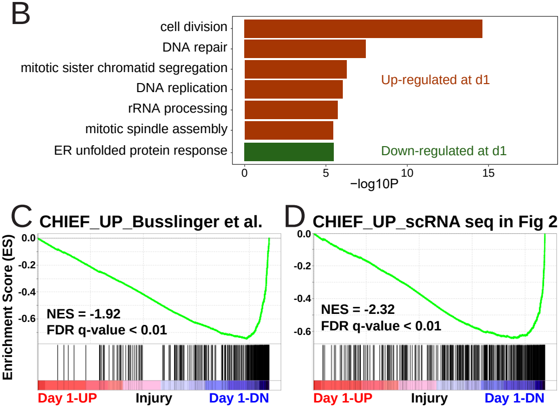

E

Source	Stange *et al.*, 2013	Leushacke *et al.*, 2017	Hata *et al.*, 2020	Busslinger *et al.*, 2021
Context	Troy+ chief cell / whole corpus	Lgr5high cell/ Lgr5- cell	Chief cell / parietal cell	Chief cell (Chia+ or Chia-) / other cells
p57 expression (log_2FC)	1.9	1.1	4.0	1.7 (Chia+); 1.5 (Chia-)

Figure S2

A

Cluster #	11	1	4	0	9	8	2	6	3	5	7	10	Total
	Chief	C/N-1	C/N-2	Neck	C/Pr-1	C/Pr-2	Pr-2	Pr-1	N/P	Pr/P-2	Pr/P-1	Pit	Total
Control	54	144	145	217	56	46	53	41	124	56	50	44	1030
Injury	11	31	7	2	39	54	110	81	32	76	68	42	553

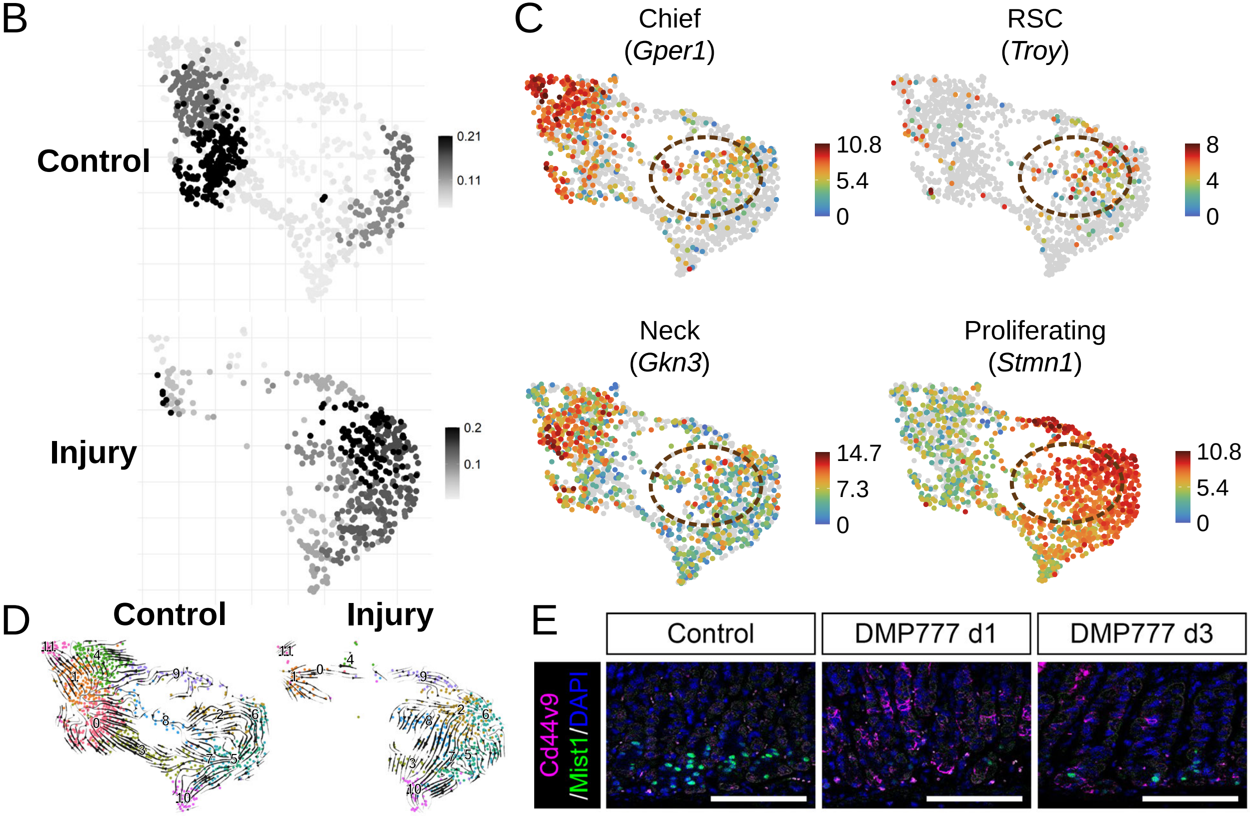

Figure S3

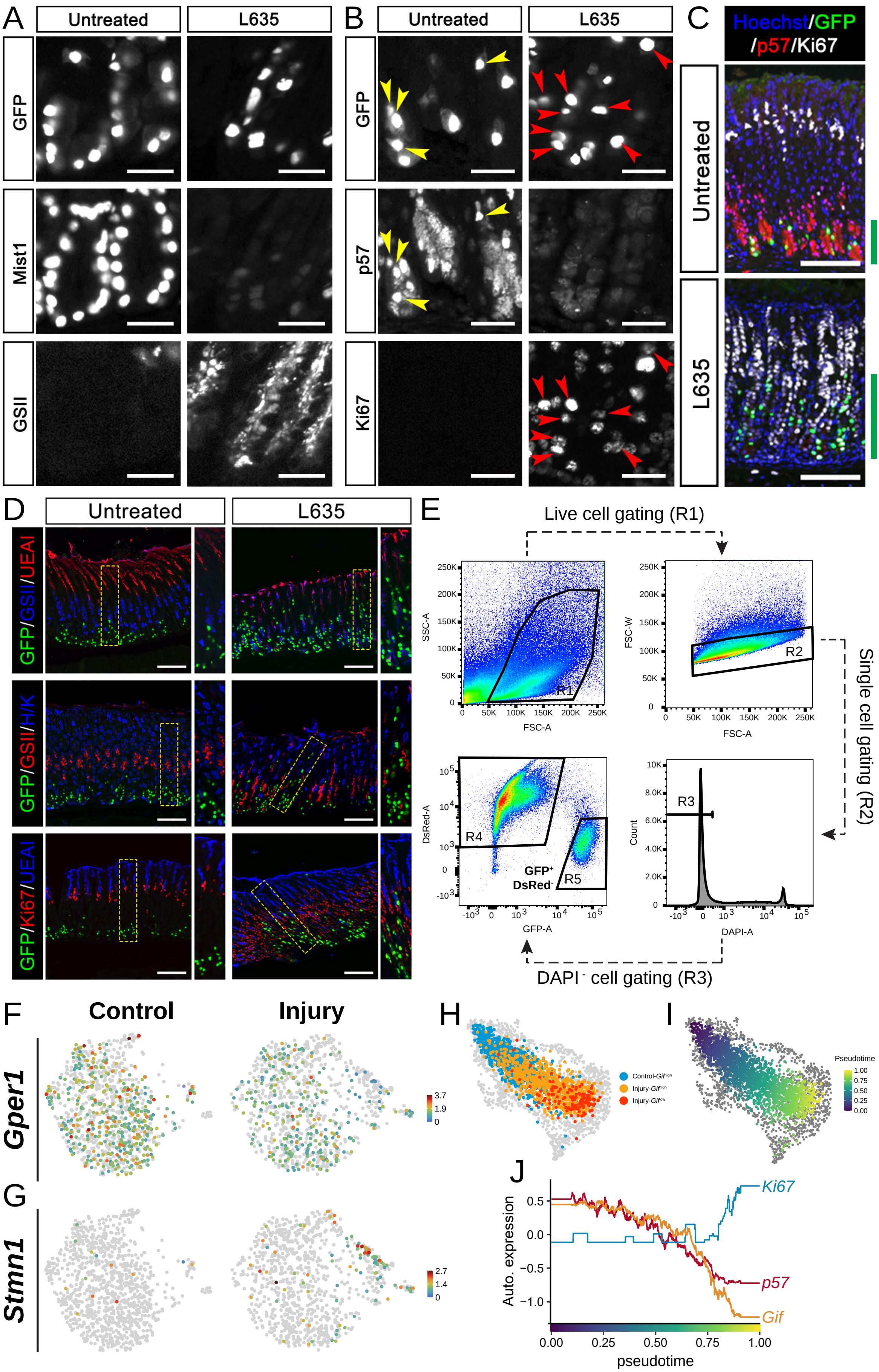

A
Untreated
L635
GFP
Mist1
GSII
B
Untreated
L635
GFP
p57
Ki67
C
Hoechst/GFP/p57/Ki67
Untreated
L635
D
Untreated
L635
GFP/GSII/UEAI
GFP/GSII/H/K
GFP/Ki67/UEAI
E
Live cell gating (R1)
Single cell gating (R2)
DAPI- cell gating (R3)
SSC-A
FSC-A
FSC-W
DsRed-A
GFP-A
Count
DAPI-A
R1
R2
R3
R4
R5
GFP+ DsRed-
F
Control
Injury
Gper1
G
Stmn1
H
Control-Gif high
Injury-Gif high
Injury-Gif low
I
Pseudotime
J
Auto. expression
pseudotime
Ki67
p57
Gif

Figure S4

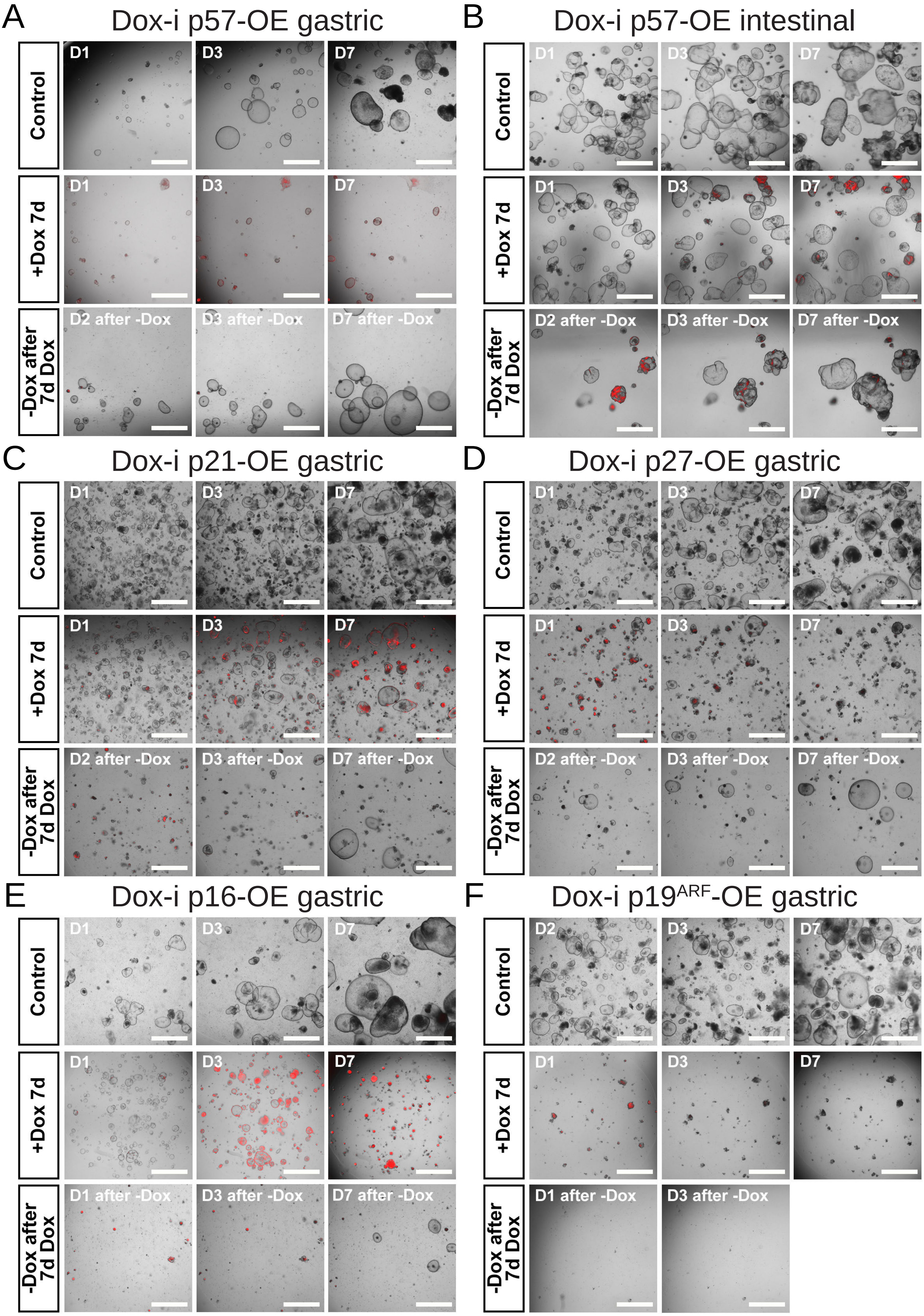
A
Dox-i p57-OE gastric
B
Dox-i p57-OE intestinal
C
Dox-i p21-OE gastric
D
Dox-i p27-OE gastric
E
Dox-i p16-OE gastric
F
Dox-i p19ARF-OE gastric
Control
+Dox 7d
-Dox after 7d Dox
D1
D2
D3
D7
D1 after -Dox
D2 after -Dox
D3 after -Dox
D7 after -Dox

Figure S5

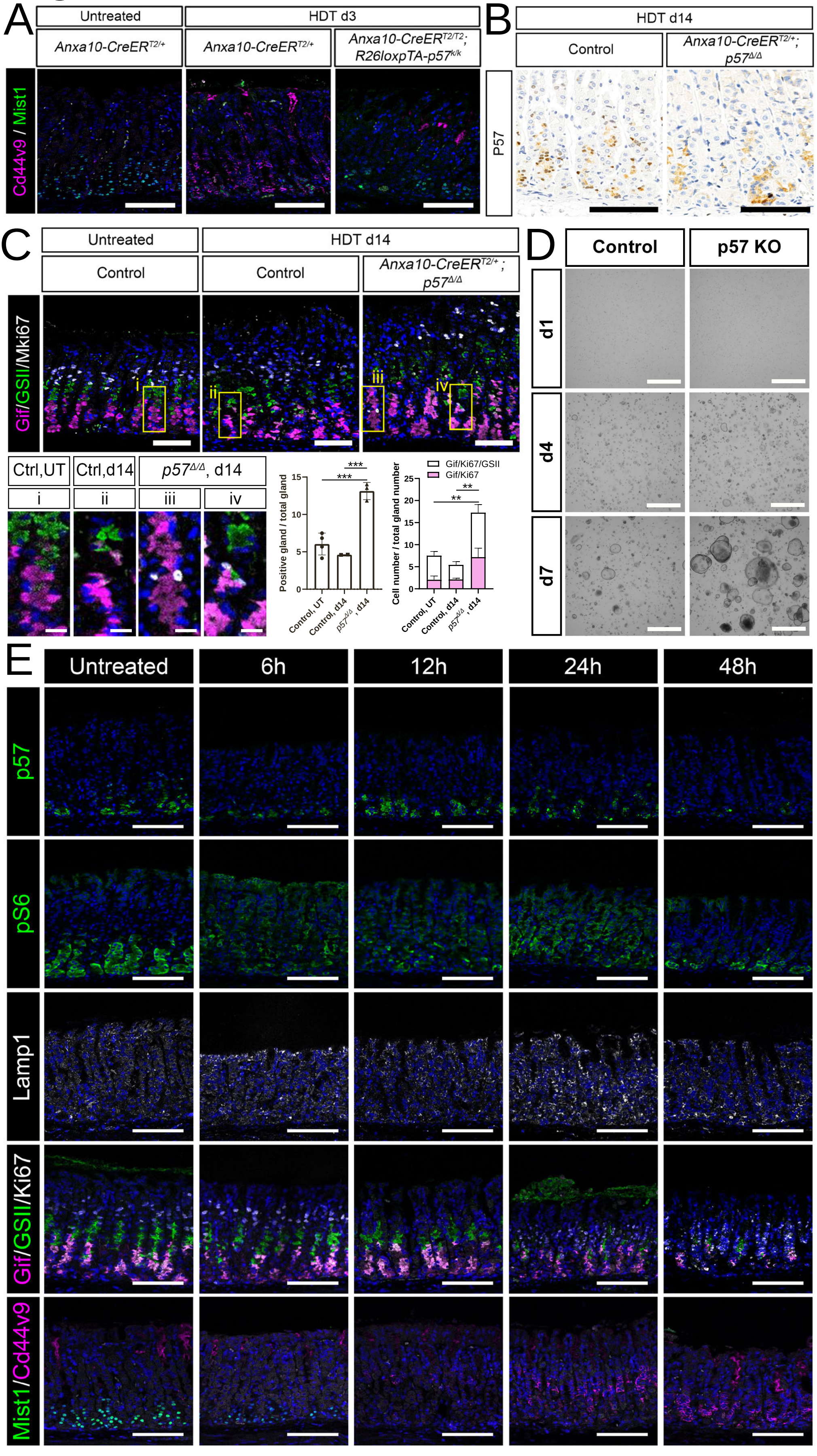
A
Untreated
HDT d3
Anxa10-CreERT2/+
Anxa10-CreERT2/+
Anxa10-CreERT2/T2; R26loxpTA-p57k/k
Cd44v9 / Mist1
B
HDT d14
Control
Anxa10-CreERT2/+; p57Δ/Δ
P57
C
Untreated
HDT d14
Control
Control
Anxa10-CreERT2/+; p57Δ/Δ
Gif/GSII/Mki67
i
ii
iii
iv
Ctrl,UT
Ctrl,d14
p57Δ/Δ, d14
Positive gland / total gland
Cell number / total gland number
Gif/Ki67/GSII
Gif/Ki67
***
**
Control, UT
Control, d14
p57Δ/Δ, d14
D
Control
p57 KO
d1
d4
d7
E
Untreated
6h
12h
24h
48h
p57
pS6
Lamp1
Gif/GSII/Ki67
Mist1/Cd44v9

Supplementary figure legends

Supplementary figure 1. Rapid transcriptome changes after injury shown in time-course bulk RNA-seq of Troy+ chief cells and enrichment of p57 expression in chief cells in homeostasis. (A) Heatmap of 1667 DEGs clustered by 6 patterns. 2-6 mice per time point were analyzed. Cluster 1 and 2 show peak expression at 1 dpi and gradual reduction during recovery. Cluster 3 and 4 show the biggest decrease in gene expression at 1 dpi. (B) Selected GO-terms enriched in upregulated (red) or downregulated (green) genes at 1 dpi (P-value < 10^{-5}). (C and D) GSEA of gene signatures associated with the chief cell signature from the scRNA-seq dataset from Busslinger et al., 2021 (C) and from the Pgc+ scRNA-seq dataset in Figure 2 (D). NES, Normalized Enrichment Score. (E) Enrichment of *p57* expression in chief cells in homeostasis from other datasets. Related to Figure 1, Tables S1 and S2.

Supplementary figure 2. Rapid switch of p57+ gastric chief cells to Ki67+ injury-responsive chief cells upon injury. (A) Cell numbers of each cluster in control and injury analyzed in Pgc+ scRNA-seq. (B) The proportion of the cell number of each cluster in control and injury samples. (C) UMAP plots of further markers for chief cells (*Gper1*), RSCs (*Troy*), neck cells (*Gkn3*), and proliferating cell markers (*Stmn1*). Brown dotted circles show injury-responsive chief cells. (D) RNA velocity inferred by scVelo projected on the UMAP plots in control and injury. (E) Double labelling of markers for SPEM (Cd44v9, magenta) and chief cells (Mist1, green) in control, at 1 dpi, and at 3 dpi. Nuclei were counterstained with DAPI (blue). Scale bars, 100 µm. Related to Figure 2.

Supplementary figure 3. Gif+ lineage tracing shows that Gif+ chief cells generate other cell types and acquire injury-responsive chief cell characteristics upon injury. (A and B) Single-channel images of Figure 3C and 3D, respectively. Scale bars, 20 µm. (C) Enlarged images of Fig 3D to show lineage tracing of Gif+ chief cells in control and injury. Green lines at the right show vertical expansion of the lineage tracing of Gif+ cells. Scale bars, 100 µm. (D) Examples of full gland lineage tracing of Gif+ chief cells (GFP). GSII, neck cell marker; UEAI, pit cell marker; H/K, H/K-ATPase, parietal cell marker. Scale bars, 100 µm. (E) Sorting strategy of Gif lineage cells (GFP^{+} $DsRed^{-}$) from *Gif-Cre-nTnG* mice. (F) UMAP plots of a further marker for chief cells (*Gper1*) in control (left) and injury (right). (G) UMAP plots of a further marker for proliferating cells (*Stmn1*) in control (left) and injury (right). (H) Projection of Gif^{high} cells in control and Gif^{high} and Gif^{low} cells in injury on the UMAP plot for Pgc+ scRNA-seq. (I) Pseudotime analysis of the Gif lineage cells in the projected UMAP of Pgc+ scRNA-seq data. (J) Gene expression trajectories along the

pseudotime trajectory. The represented expression values are log2-transformed normalized read counts followed by the z transform. The pseudotime is denoted in the bar on the x axis. The expression of *Ki67* gets increased while the expression of *p57* and *Gif* gets decreased along the pseudotime trajectory. Related to Figure 3.

Supplementary figure 4. Regrowth assay after expressing cell cycle inhibitors in gastric organoids and p57 in intestinal organoids by Dox-inducible system. (A and B) 7d of Dox treatment and Dox withdrawal experiments in Dox-i p57-OE gastric organoids (A) and in Dox-i p57-OE intestinal organoids (B). (C and D) 7d of Dox treatment and Dox withdrawal experiments in Dox-inducible CIP/KIP family CKI expression. (C) Dox-i p21-OE gastric organoids. (D) Dox-i p27-OE gastric organoids. (E) 7d of Dox treatment and Dox withdrawal experiments in Dox-inducible expression of INK4 family of CKI, p16 in gastric organoids. (F) 7d of Dox treatment and Dox withdrawal experiments in Dox-inducible expression of $p19^{ARF}$ in gastric organoids. Scale bars, 1 mm. Related to Figure 4.

Supplementary figure 5. Injury response is inhibited in p57 OE and prolonged in p57 KO *in vivo*. (A) Double staining of markers for SPEM (Cd44v9, magenta) and chief cells (Mist1, green) in the conditions as outlined above. Scale bars, 100 µm. (B) p57 staining in control and p57 knockout epithelium after 14 d of HDT treatment. Scale bars, 100 µm. (C) Triple staining with markers for chief cells (Gif, magenta), neck cells (GSII, green), and proliferating cells (Ki67, white) and quantification of injury responsive chief cells. 2-4 mice per condition were analyzed. Scale bars, 100 µm for the upper figure sets, 20 µm for the insets. Data in the graphs are represented as mean ± SD. **P<0.01 and ***P< 0.001 calculated by one-way ANOVA. (D) Growth difference between control and p57 knockout organoids. Scale bars, 1 mm. (E) Labelling with several markers for the metabolic and molecular changes of chief cell transition upon injury. Scale bars, 100 µm. Related to Figure 5.
