# Supplementary material for: p57Kip2 imposes the reserve stem cell state of gastric chief cells
Source: Cell Stem Cell. 2022 May 5;29(5):826–839.e9. doi: 10.1016/j.stem.2022.04.001 (PMC9097776; doi:10.1016/j.stem.2022.04.001)
Supplement: Document S2. Article plus supplemental information [file mmc5.pdf]

Cell Stem Cell

Article

p57Kip2 imposes the reserve stem cell state of gastric chief cells

Graphical abstract

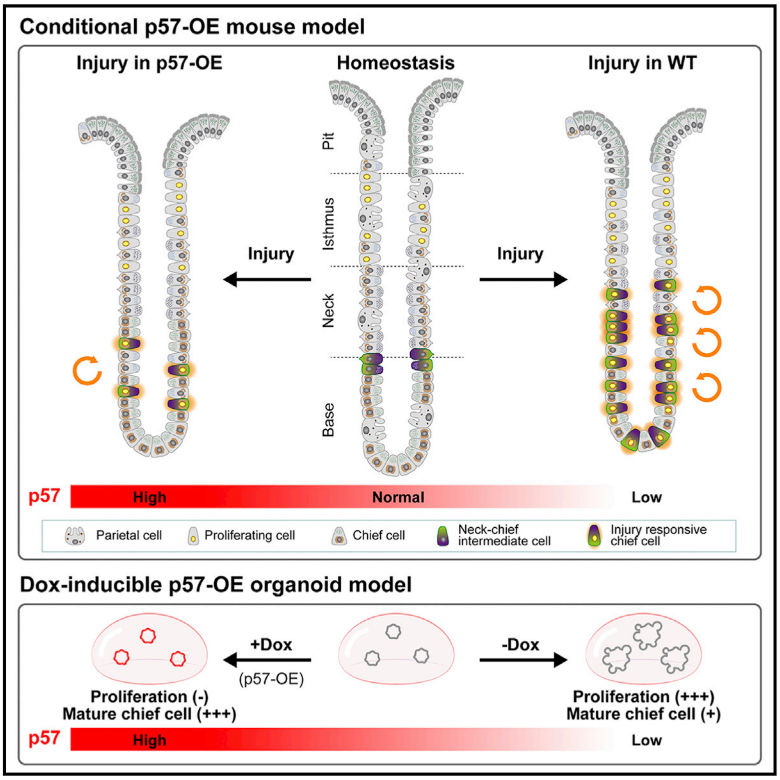

Authors

Ji-Hyun Lee, Somi Kim, Seungmin Han, ..., Eunyoung Choi, Bon-Kyoung Koo, Jong Kyoung Kim

Correspondence

eunyoung.choi@vumc.org (E.C.), koobk@ibs.re.kr or koobk@ibs.re.kr (B.-K.K.), blkimjk@postech.ac.kr (J.K.K.)

In brief

The teams of Kim, Koo, and Choi show that p57 acts as a molecular switch governing the injury response of gastric chief cells. Upon injury, chief cells lose p57 expression for rapid proliferation and tissue regeneration. The p57 expression imposes the reserve stem cell state of chief cells in homeostasis.

Highlights

- Gastric chief cells undergo rapid transcriptional changes during injury repair
- p57+ chief cells rapidly switch to distinct injury-responsive chief cells upon injury
- p57 induces RSC-like state in gastric corpus organoids
- p57 expression inhibits the induction of injury-responsive chief cells *in vivo*

Lee et al., 2022, Cell Stem Cell *29*, 826–839
May 5, 2022 © 2022 The Authors. Published by Elsevier Inc.
https://doi.org/10.1016/j.stem.2022.04.001

CellPress
OPEN ACCESS

Cell Stem Cell

Article

p57^{Kip2} imposes the reserve stem cell state of gastric chief cells

Ji-Hyun Lee,[1,14] Somi Kim,[2,13,14] Seungmin Han,[3,4,14] Jimin Min,[5,6,15] Brianna Caldwell,[5,6,15] Aileen-Diane Bamford,[1,15] Andreia Sofia Batista Rocha,[1,15] JinYoung Park,[1,15] Sieun Lee,[1,15] Szu-Hsien Sam Wu,[1] Heetak Lee,[1] Juergen Fink,[3] Sandra Pilat-Carotta,[1] Jihoon Kim,[1,7] Manon Josserand,[3] Réka Szep-Bakonyi,[1] Yohan An,[8] Young Seok Ju,[8] Anna Philpott,[3,9] Benjamin D. Simons,[3,4,10] Daniel E. Stange,[11] Eunyoung Choi,[5,6,*] Bon-Kyoung Koo,[1,12,16,*] and Jong Kyoung Kim[2,13,*]

[1]Institute of Molecular Biotechnology of the Austrian Academy of Sciences (IMBA), Vienna Biocenter (VBC), Dr. Bohr-Gasse 3, Vienna, 1030, Austria
[2]Department of Life Sciences, Pohang University of Science and Technology (POSTECH), Pohang 37673, Republic of Korea
[3]Wellcome Trust/Medical Research Council Cambridge Stem Cell Institute, Jeffrey Cheah Biomedical Centre, University of Cambridge, Cambridge CB2 0AW, UK
[4]Wellcome Trust/Cancer Research UK Gurdon Institute, University of Cambridge, Cambridge CB2 1QN, UK
[5]Department of Surgery and Epithelial Biology Center, Vanderbilt University Medical Center, Nashville, TN, USA
[6]Cell and Developmental Biology, Vanderbilt University School of Medicine, Nashville, TN, USA
[7]Department of Medical and Biological Sciences, The Catholic University of Korea, Bucheon, Gyeonggi-do, Republic of Korea
[8]Graduate School of Medical Science and Engineering, Korea Advanced Institute of Science and Technology (KAIST), Daejeon 34141, Republic of Korea
[9]Department of Oncology, University of Cambridge, Hutchison/MRC Research Centre, Cambridge Biomedical Campus, Cambridge CB2 0XZ, UK
[10]Department of Applied Mathematics and Theoretical Physics, Centre for Mathematical Sciences, University of Cambridge, Wilberforce Road, Cambridge CB3 0WA, UK
[11]Department of Visceral, Thoracic and Vascular Surgery, University Hospital Carl Gustav Carus, Medical Faculty, Technische Universität Dresden, Fetscherstr. 74, 01307 Dresden, Germany
[12]Center for Genome Engineering, Institute for Basic Science, 55, Expo-ro, Yuseong-gu, Daejeon 34126, Republic of Korea
[13]Department of New Biology, DGIST, Daegu 42988, Republic of Korea
[14]These authors contributed equally
[15]These authors contributed equally
[16]Lead contact
*Correspondence: eunyoung.choi@vumc.org (E.C.), koobk@ibs.re.kr or koobk@ibs.re.kr (B.-K.K.), blkimjk@postech.ac.kr (J.K.K.)
https://doi.org/10.1016/j.stem.2022.04.001

SUMMARY

Adult stem cells constantly react to local changes to ensure tissue homeostasis. In the main body of the stomach, chief cells produce digestive enzymes; however, upon injury, they undergo rapid proliferation for prompt tissue regeneration. Here, we identified p57^{Kip2} (p57) as a molecular switch for the reserve stem cell state of chief cells in mice. During homeostasis, p57 is constantly expressed in chief cells but rapidly diminishes after injury, followed by robust proliferation. Both single-cell RNA sequencing and dox-induced lineage tracing confirmed the sequential loss of p57 and activation of proliferation within the chief cell lineage. In corpus organoids, p57 overexpression induced a long-term reserve stem cell state, accompanied by altered niche requirements and a mature chief cell/secretory phenotype. Following the constitutive expression of p57 *in vivo*, chief cells showed an impaired injury response. Thus, p57 is a gatekeeper that imposes the reserve stem cell state of chief cells in homeostasis.

INTRODUCTION

As in humans, the mouse gastric corpus is the main body of the stomach with numerous glandular units that secrete digestive enzymes and acid. The gastric gland consists of four parts: pit, isthmus, neck, and base, starting from the lumen. In the corpus glands, stem cell populations reside in two distinct regions (Burclaff et al., 2020; Han et al., 2019). The isthmus contains rapidly cycling Ki67- and Stmn1-expressing stem cells (isthmus stem cells [IsthSCs]), which constantly replenish the gland from pit to neck (Han et al., 2019). Although the exact identity of quiescent IsthSCs is still elusive, various markers have been proposed (Arnold et al., 2011; Choi et al., 2018). At the base, a second Troy- and Lgr5-expressing stem cell population (base stem cells [BSCs]) resides (Leushacke et al., 2017; Stange et al., 2013). These BSCs are slow cycling in homeostasis and serve as chief

 © 2022 The Authors. Published by Elsevier Inc.
This is an open access article under the CC BY license (http://creativecommons.org/licenses/by/4.0/).

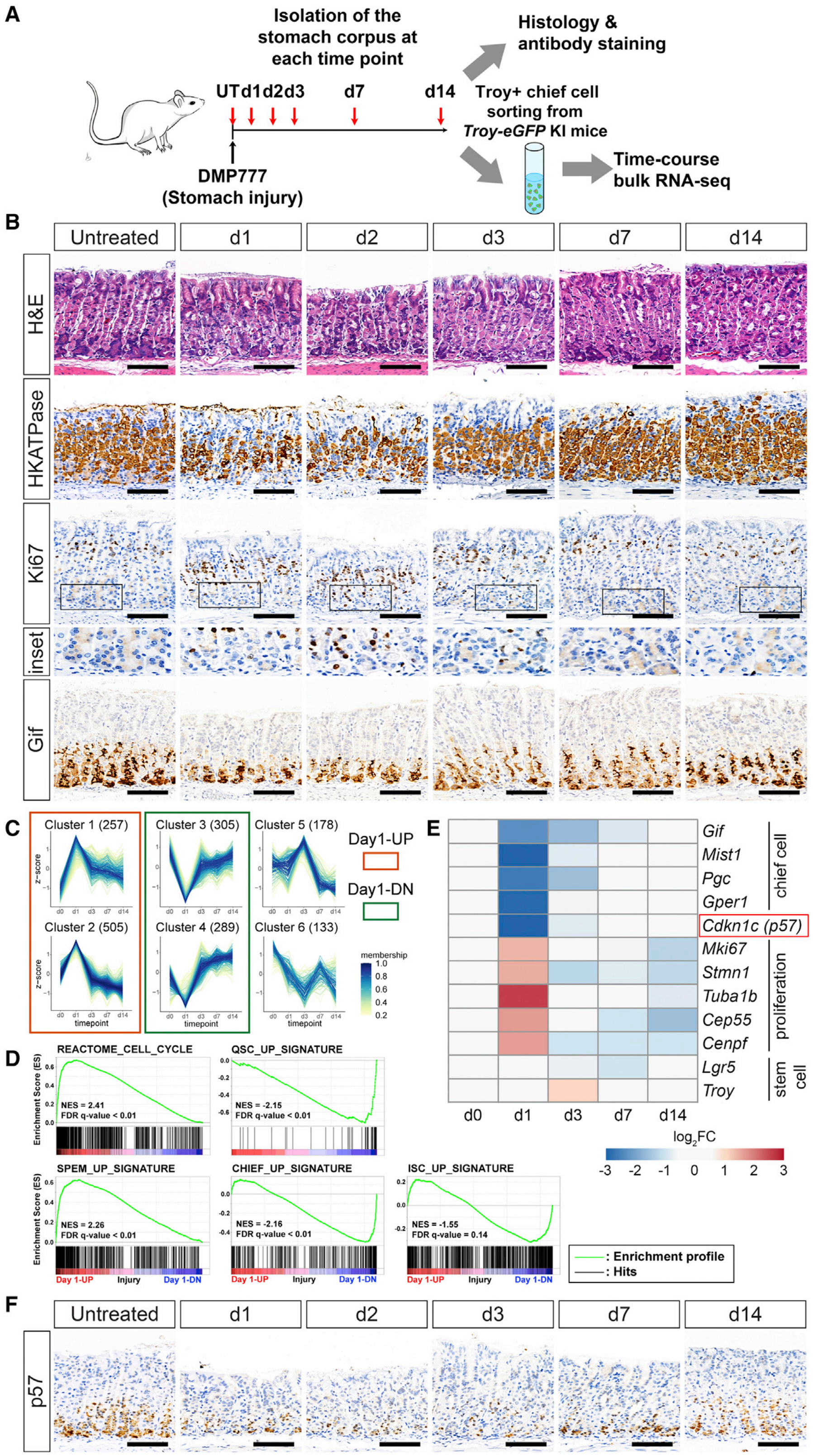

Figure 1. Time course bulk RNA-seq of Troy+ chief cells after injury shows a rapid change in transcriptome including downregulation of p57, a candidate for chief cell activation

(A) Experimental scheme for time course analysis of the corpus epithelium and bulk-RNA seq of Troy+ cells (GFP positive) upon damage. UT, untreated.

(legend continued on next page)

cells that secrete zymogens (Dieckgraefe et al., 1988; Nam et al., 2010).

Chief cells are referred to as "reserve stem cells (RSCs)" since they become proliferative and take part in regeneration following damage to the stomach epithelium (Leushacke et al., 2017; Stange et al., 2013). Experiments using Troy+ and Lgr5+ lineage tracing together with the depletion of specific cell populations by chemical injury—e.g., proliferative cells by fluorouracil (5-FU), acid-secreting parietal cells by proton pump inhibitors such as DMP-777 and L635 (Nam et al., 2010; Nomura et al., 2005), or indeed global tissue damage by high-dose tamoxifen (HDT) (Huh et al., 2012)—have shown that chief cells (BSCs and RSCs) rapidly proliferate to differentiate into all the different cell lineages of the stomach epithelium (Leushacke et al., 2017; Nam et al., 2010; Stange et al., 2013). Nonetheless, these observations have been challenged, since tamoxifen-based lineage tracing itself can induce tissue damage, complicating the interpretation of the lineage tracing results (Hata et al., 2020). In addition, the underlying molecular mechanisms that control the chief cell behavior in homeostasis and injury repair are not yet fully understood. Previous reports have shown the involvement of the mTOR, p53, and lysosomal pathways (Miao et al., 2020; Willet et al., 2018), but a key molecular switch that mediates injury-mediated signal activation to downstream responses has not been identified.

Here, we reveal $p57^{Kip2}$ (p57, also known as Cdkn1c) as a molecular switch that regulates the plastic behavior of chief cells in homeostasis and injury repair. Normally, chief cells express high levels of p57 but rapidly lose expression of p57 by 1 day after injury. These chief cells then adopt a unique spasmolytic polypeptide-expressing metaplasia (SPEM) cell-like state, which we call "injury-responsive chief cells," expressing multiple markers of chief cells, neck cells, proliferating cells, and stem cells, as shown in previous studies (Burclaff et al., 2020; Caldwell et al., 2021; Leushacke et al., 2017; Miao et al., 2020; Nam et al., 2010; Nozaki et al., 2008; Stange et al., 2013; Willet et al., 2018). In gastric corpus organoids, where stem cells are proliferative, p57 expression reinstates the RSC state with altered niche requirements and mature chief cell characteristics. When constitutive expression of p57 is introduced *in vivo*, chief cells retain a nonproliferative chief cell phenotype even after tissue injury. Our data confirm the RSC characteristics of chief cells with single-cell RNA sequencing (scRNA-seq) and doxycycline (Dox)-based lineage tracing. These also identify the existence of a critical regulatory switch in gastric chief cells that allows them to alternate between homeostatic chief cell and injury-responsive chief cell states.

RESULTS

Troy+ chief cells undergo rapid transcriptional changes during injury repair

To profile the molecular changes associated with chief cells in the course of injury repair, we first used immunohistochemistry to characterize the injury response in corpus sections taken at 1, 2, 3, 7, and 14 days after DMP-777-mediated injury, as well as non-treated controls (Figures 1A and 1B). As previously reported, a single dose of DMP-777 led to an acute depletion of acid-secreting parietal cells (Nomura et al., 2005) at 1 day postinjury (dpi), and the parietal cells were fully recovered by 14 dpi (Figure 1B, H/K-ATPase). At 2 and 3 dpi, the proliferation marker Ki67 was highly upregulated at the base to levels comparable with those seen in the isthmus, then rapidly diminished by 7 dpi (Figure 1B), indicating that the cells at the base are activated upon injury, as previously described (Leushacke et al., 2017; Nam et al., 2010; Stange et al., 2013). Expression of the chief cell marker Gif was also downregulated immediately after injury and then slowly recovered to homeostatic levels by 14 dpi (Figure 1B), suggesting that the injury not only activates proliferative responses but also suppresses the chief cell phenotype at the base.

To identify detailed molecular changes associated with injury-responsive chief cells in the course of injury repair, we performed time course bulk RNA sequencing (RNA-seq) analysis on sorted Troy+ chief cells from *Troy-eGFP* knockin (KI) mice (Figure 1A). We identified 1,667 differentially expressed genes (DEGs) that showed an expression change of more than 2-fold (adjusted p value < 0.01) at one or more time points compared with Troy+ chief cells from untreated controls (Figure S1A; Table S1). Among 6 identified gene expression patterns (Figure 1C), we focused on four clusters (red and green rectangles) whose expression changed dramatically at 1 dpi. These four clusters contained the majority of DEGs (1,356 genes), indicating that most gene expression changes in Troy+ chief cells occur as early as 1 day after injury, before the hyperproliferation that was detected at the protein level at 2–3 dpi (Figures 1B and 1C). Gene set enrichment analysis (GSEA) (Mootha et al., 2003; Subramanian et al., 2005) and gene ontology biological processes (GOBPs) enrichment analysis showed that Troy+ chief cells are rapidly converted to an active proliferative state upon tissue damage. The upregulated genes at 1 dpi showed a significant enrichment for genes upregulated in SPEM cells (Nozaki et al., 2008) as well as genes in a cell proliferation-related gene set and related GO terms (Figures 1D, 1E, and S1B). Downregulated genes at 1dpi showed high enrichment for genes commonly upregulated in quiescent stem cells (QSCs) (quiescent hematopoietic stem cells, muscle stem cells, and hair follicle stem cells) (Cheung and Rando, 2013) and genes expressed in gastric chief

(B) Immunohistochemistry of the stomach corpus epithelium upon DMP-777-induced injury, representative of 2–4 mice used per time point. Scale bars, 100 μm.
(C) Expression profiles of 6 clusters from the time course bulk RNA-seq. Membership values indicate the degree to which data points belong to a cluster. The number of genes of each cluster is indicated in the brackets.
(D) GSEA of gene signatures associated with the cell cycle, SPEM, quiescent stem cells (QSCs), chief cells, and intestinal stem cells (ISCs). NES, normalized enrichment score.
(E) Heatmap of the marker genes and a candidate (red rectangle) molecular switch for activation of reserve stem cells. Expression of the stemness genes was stable during the injury response.
(F) Expression pattern of p57 upon DMP-777-induced injury. Scale bars, 100 μm.
See also Figure S1 and Tables S1 and S2.

CellPress
OPEN ACCESS

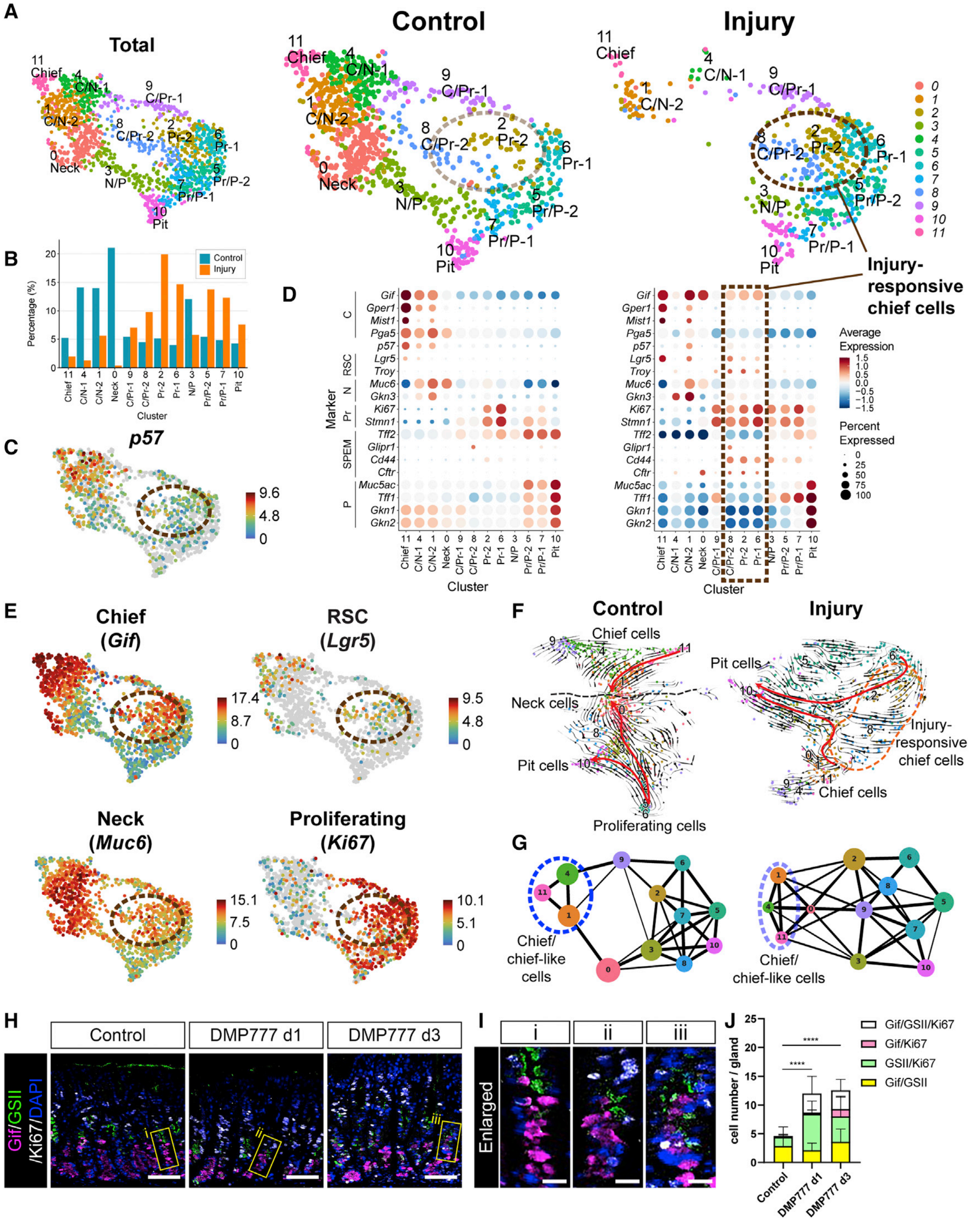

Figure 2. Single-cell transcriptomic analysis identifies a specific injury-responsive chief cell population that appears after injury of the stomach epithelium

(A) UMAP plots of scRNA-seq from Pgc+ cells in both conditions (Total, left), uninjured (Control, middle), and 1 day after DMP-777-induced corpus injury (Injury, right). Twelve clusters are annotated as follows. 0, Neck; 1, C/N-2; 2, Pr-2; 3, N/P; 4, C/N-1; 5, Pr/P-2; 6, Pr-1; 7, Pr/P-1; 8, C/Pr-2; 9, C/Pr-1; 10, Pit; 11, Chief. Full names are provided in the results section. Brown-dotted circles show where the injury-responsive chief cell population is mapped in control (faint brown) and injury (vivid brown) conditions.

(B) Percentage of cells in each cluster per condition.

(C) UMAP plot of *p57* expression.

(D) Dot plots for expression of marker genes of each cell type in control (left) and injury (right) conditions. The box indicates the injury-responsive chief cell population.

(legend continued on next page)

cells from three different reference sets (Busslinger et al., 2021 for Figure S1C; Hata et al., 2020 for Figure 1D; Pgc+ scRNA-seq from Figure 2 for Figure S1D) (Figures 1D, 1E, S1C, and S1D; Table S2). On the other hand, the intestinal stem cell (ISC) signature genes (Muñoz et al., 2012) showed no significant enrichment (Figure 1D; Table S2), and markers of RSCs such as Troy and Lgr5 did not change more than 2-fold in either direction (Figure 1E), indicating that the expression patterns of stem cell-related genes are fairly stable in gastric chief cells during injury repair.

Among the significantly downregulated genes, we were particularly interested in *p57* (from cluster 3) as a candidate switch for the activation of chief cells upon injury, since its function and expression pattern would fit very well with the injury response of Troy+ chief cells. p57 is a cyclin-dependent kinase (Cdk) inhibitor (CKI) that negatively regulates cell cycle progression by blocking cyclin/Cdk complexes. p57 belongs to the KIP/CIP class of CKIs together with p21 and p27, mainly inhibiting G1-S transition (Liu et al., 2019). Using immunohistochemistry, we confirmed that p57 is highly expressed only in the chief cell compartment at the base in homeostatic conditions and is dramatically reduced from 1 dpi (Figure 1F), which is quickly followed by the proliferative response in the base, which was observed as early as 2 dpi (as shown in Figure 1B, Ki67). We also confirmed that expression of *p57* was commonly enriched in chief cells from several transcriptomic analyses (Busslinger et al., 2021; Hata et al., 2020; Leushacke et al., 2017; Stange et al., 2013; Figure S1E).

p57+ gastric chief cells rapidly switch to Ki67+ injury-responsive chief cells upon injury

As the Troy+ chief cells (Troy+ RSCs) showed a rapid transition from p57+ RSCs to Ki67+ injury-responsive cells, we decided to assess the overall injury response of gastric chief cells, as well as other cell types, via single-cell transcriptomic analysis using the plate-based full-length scRNA-seq method. We compared the transcriptomes of Pgc+ gastric corpus cells from *Pgc-DsRed* KI mice during homeostasis and 1 dpi after DMP-777 treatment. As we reported previously, *Pgc* was broadly expressed in different gastric epithelial cell types, except for parietal cells and enteroendocrine cells, although it is known as a chief cell marker (Han et al., 2019). To compare transcriptomic changes of cell types between normal and injured tissues, 1,583 high-quality cells from the two conditions were visualized in a uniform manifold approximation and projection (UMAP) plot (Figure 2A). We identified 12 distinct clusters of epithelial cells by unsupervised clustering and annotated them based on marker gene expression (Figures 2A–2E). Apart from the major cell types already known in the stomach corpus glands, such as chief (C, cluster 11 (C11)), neck (N, C0), proliferating progenitor (Pr-1, Pr-2; C6, C2, respectively), and pit cells (P, C10), several intermediate populations were found such as chief/neck intermediates (C/N-1, C/N-2; C4, C1, respectively), neck/pit intermediates (N/P, C3), chief-proliferating progenitors (C/Pr-1, C/Pr-2; C9, C8, respectively), and proliferating progenitor/pit intermediates (Pr/P-1, Pr/P-2; C7, C5, respectively), which were named based on the location of each cluster in the UMAP.

The cell clusters underwent dramatic changes between control and injury. In homeostasis, C; C/N-1, 2; and N cells were abundant, whereas after injury, their numbers were substantially decreased (Figures 2A, 2B, S2A, and S2B). *p57* expression closely overlapped with the entire *Gif*+ chief cell population in homeostasis (Figure 2C). In injury, instead, we found a newly emerging cell population, including C/Pr-2, Pr-2, and partly Pr-1 clusters, which shows a greatly reduced *p57* expression (Figures 2A and 2C). Unlike the cells in the corresponding clusters during homeostasis, the injury-induced cells expressed the RSC markers *Troy* and *Lgr5* (Figure 2D). We named these cells "injury-responsive chief cells" (Figures 2A and 2D). As this population appears within 1 dpi, which is not enough time for generating an entirely new population of cells, we presume that they represent an altered state of pre-existing chief cells. Interestingly, we found that these injury-responsive chief cells coexpressed the marker genes of chief cells (*Gif* and *Gper1*) (Hata et al., 2020), neck cells (*Muc6* and *Gkn3*) (Menheniott et al., 2010), proliferating cells (*Ki67* and *Stmn1*), and RSCs (*Lgr5* and *Troy*)—a characteristic of uncommitted progenitor cells (Figures 2E and S2C). Besides, when we applied RNA velocity (Bergen et al., 2020; La Manno et al., 2018) and partition-based graph abstraction (PAGA) analysis, we found that chief cells (C11) are connected and differentiated to C/Pr-2 (C8, injury responsive), Pr-2 (C2, injury responsive), and further to pit (C10) cells (Figures 2F, 2G, and S2D). In contrast, we found a clear separation between chief cell-related clusters (C1, 4, 11) and the remaining clusters in homeostasis (Figures 2F and 2G), which fits well with previous observations that the base and the rest of the gland are two separate compartments in homeostasis (Burclaff et al., 2020; Han et al., 2019). Lastly, we used immunostaining to confirm the emergence of injury-responsive progenitors not committed to any of the differentiated cell fates. We found that the number of cells coexpressing at least two different markers of chief cells (Gif), neck cells (GSII), or

(E) UMAP plots showing marker gene expression of chief cells, RSCs, neck cells, and proliferating cells. Brown-dotted circles indicate the injury-responsive chief cell population.

(F) t-distributed stochastic neighbor embedding (t-SNE) plots showing RNA velocity inferred by scVelo in control and injury conditions. Injury-responsive chief cells are located in the orange-dotted area.

(G) PAGA graph showing all the edges connecting cell clusters (nodes) in control and injury conditions. The width of the edges quantifies the connectivity between clusters. Blue-dotted circles indicate chief or chief-like cells in control (vivid) and injury (faint). Topological modularities (Q) are 0.14 and 0.06 in the control and injury samples, respectively.

(H) Triple staining of markers for chief cells (Gif, magenta), neck cells (GSII, green), proliferating cells (Ki67, white) in control, at 1 dpi and at 3 dpi. Nuclei were counterstained with DAPI (blue). Scale bars, 100 μm.

(I) Insets of figure (H). Scale bars, 20 μm.

(J) Quantification of injury-responsive cells ($Gif^+/GS\text{-}II^+$, $Gif^+/Ki67^+$, $GS\text{-}II^+/Ki67^+$, and $Gif^+/GS\text{-}II^+/Ki67^+$). Twenty glands from 2–3 mice per condition were analyzed. Data are represented as mean ± SD. ****$p < 0.0001$ calculated by one-way ANOVA for total injury-responsive cell number.

See also Figure S2.

CellPress
OPEN ACCESS

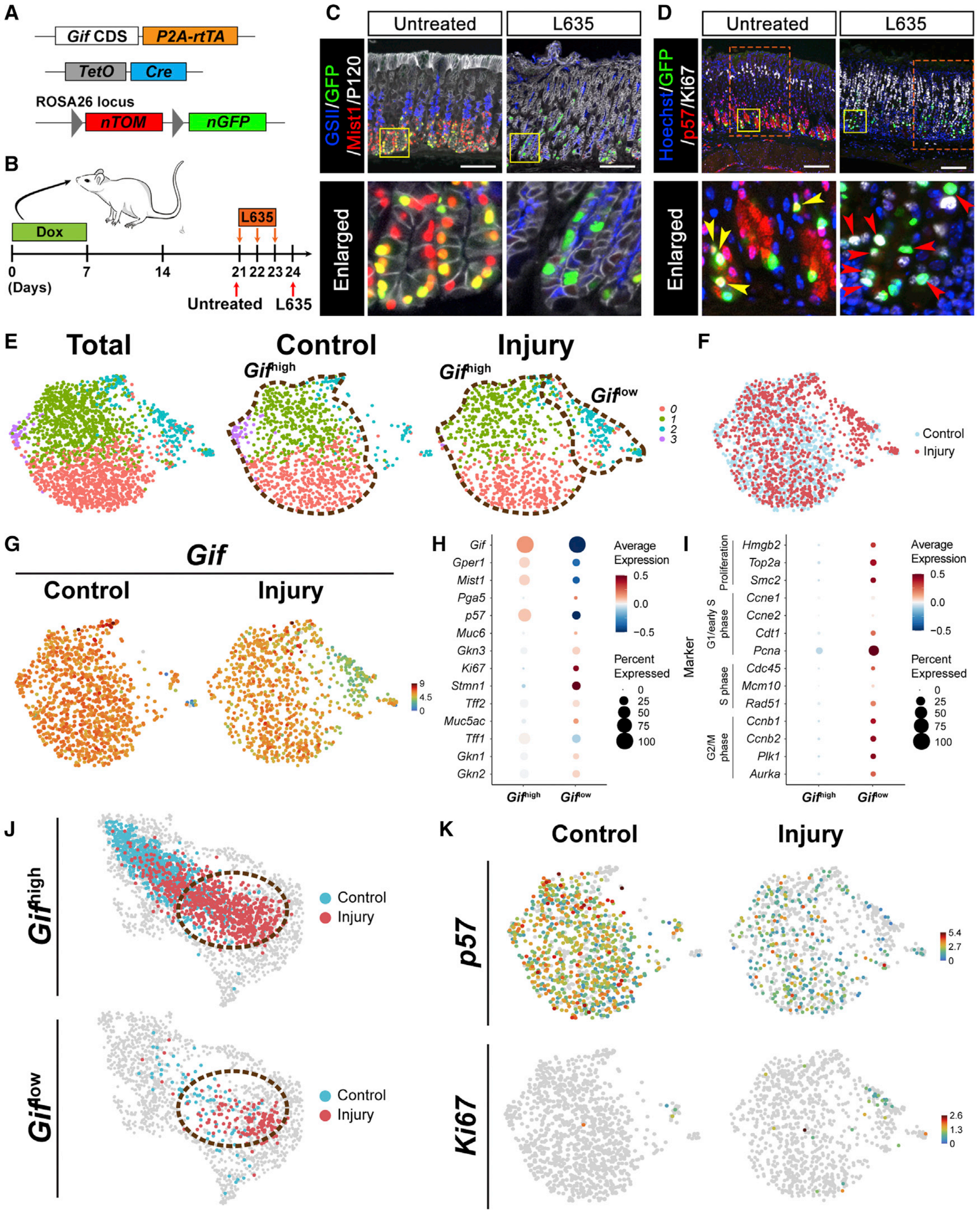

Figure 3. Lineage tracing and scRNA-seq by Gif+ chief cells identify injury-responsive chief cells

(A) Diagram of the *Gif-Cre-nTnG* allele used in this study.

(B) Experimental scheme of the L635 treatment study in *Gif-Cre-nTnG* mice. Dox was administered for 1 week and off for 2 weeks to label chief cells with GFP. The mice were then left untreated or treated with 3 doses of L635.

(C and D) Paraffin sections of *Gif-Cre-nTnG* mouse stomachs untreated or treated with 3 doses of L635 were immunolabeled with antibodies against (C) GFP (green), Mist1 (chief cells, red), GSII-lectin (neck cells, blue), and P120 (epithelial cell membrane, white) or (D) GFP (green), p57 (red), and Ki67 (proliferating cells, white). GFP+Mist1+ cells indicate chief cells and GFP+GSII+ cells indicate injury-responsive chief cells. Arrowheads indicate GFP+p57+ double-positive cells (yellow) and GFP+Ki67+p57-proliferating injury-responsive chief cells (red), respectively. Nuclei were counterstained with hoechst (blue) in (D). Yellow boxes indicate enlarged areas. Orange-dotted boxes indicate enlarged areas shown in Figure S3C. Scale bars, 100 μm.

(E) UMAP plots of scRNA-seq from Gif lineage cells ($GFP^{+}TdTom^{-}$) of *Gif-Cre-nTnG* mice in both conditions (Total, left), uninjured (Control, middle), and 2 days after DMP-777-induced corpus injury (Injury, right). Gif^{high} and Gif^{low} populations are marked by brown-dotted lines.

(legend continued on next page)

proliferative cells (Ki67) was increased in the base region upon injury (Figures 2H–2J). Additionally, we also confirmed that expression of a SPEM marker (Cd44v9) was increased, whereas expression of another chief cell marker Mist1 was almost lost upon injury (Figure S2E).

Injury-responsive chief cells are derived from Gif+ chief cells

To prove the direct lineage relationship between chief cells and injury-responsive chief cells, we performed lineage tracing using *Gif-rtTA* mice (Caldwell et al., 2021). This allows us to perform clean lineage tracing in the stomach using a Dox-inducible reporter system without risking possible damage by tamoxifen. We combined *Gif-rtTA* with *TetO-Cre* and *Rosa26-nTnG* lines (*Gif-Cre-nTnG*; Figure 3A). We then administrated Dox in drinking water for 1 week to label Gif+ chief cells. After 2 weeks, we treated with L635 3 times for 3 days to induce an injury response. Mice were sacrificed within a day after the last treatment (Figure 3B). As we hypothesized from both the bulk RNA-seq and scRNA-seq data, we observed that labeled chief cells (GFP+) underwent a rapid loss of chief cell markers (Mist1 and p57) and became proliferative (Ki67) as well as GSII positive (Figures 3C, 3D, S3A, and S3B). We also found that during the acute injury response, the lineage tracing of Gif+ chief cells extended upward, usually to more than half of the gland and sometimes even to the full gland, showing that the Gif+ chief cells contribute to the regeneration of the whole gland (Figures S3C and S3D).

Next, we performed scRNA-seq coupled with Dox-mediated Gif lineage tracing using the *Gif-Cre-nTnG* mouse model to confirm that the injury-responsive chief cell population defined from the previous scRNA-seq using Pgc+ cells was indeed derived from chief cells. After labeling chief cells with GFP by Dox administration to *Gif-Cre-nTnG* mice, we waited for 3 weeks for maturation of labeled chief cells. We sorted GFP^{+} $tdTom^{-}$ cells at 2 dpi or without injury as a control (Figure S3E) to cell-capture plates and performed scRNA-seq using the SORT-seq method (van den Brink et al., 2017; Muraro et al., 2016). We identified 4 clusters by unsupervised clustering (Figures 3E and 3F), which were then classified into Gif^{high} and Gif^{low} subpopulations based on the expression of *Gif* (Figures 3E, 3G, and 3H). As expected, control cells were mostly Gif^{high}, whereas Gif^{low} cells appear after injury (Figures 3E and 3G). Gif^{low} cells showed various markers for cell cycle (Figure 3I).

When we projected the Gif lineage scRNA-seq data to the previous UMAP of Pgc+ scRNA-seq, we found that both Gif^{high} and Gif^{low} cells from 2 dpi fell into the previously defined injury-responsive chief cell domain (Figure 3J), whereas the Gif^{high} cells from the control condition overlapped well with the chief cell domain (Figure 3J). This suggests that even the Gif^{high} cells in the injury condition have undergone transcriptional changes to become injury-responsive chief cells. Accordingly, the Gif^{high} cells at 2 dpi show a lower expression level of *Gif*, *Gper1*, and *p57* (Figures 3G, 3K, and S3F) compared with the Gif^{high} cells from the uninjured control. The Gif^{low} cells at 2 dpi show no or reduced expression of *Gif*, *Gper1*, and *p57* (Figures 3G, 3K, and S3F), but instead, they start expressing proliferation markers, *Ki67* and *Stmn1* (Figures 3K and S3G–S3J).

Taken together, our data show strong plasticity of p57+ gastric chief cells, which rapidly switch their state to Ki67+ injury-responsive chief cells upon injury in order to produce other cell types and promote repair, consistent with earlier predictions from tamoxifen-based lineage tracing (Leushacke et al., 2017; Nam et al., 2010; Stange et al., 2013).

p57 induces a reserve stem cell-like state in gastric corpus organoids

As p57 expression showed a tight negative correlation with the proliferative response of chief cell-derived injury-responsive cells during injury repair, we decided to assess its function using gastric corpus organoids. Corpus organoids represent the rapidly expanding base-neck compartment with proliferating progenitors, chief cells, and neck cells (Bartfeld et al., 2015; Leushacke et al., 2017; Stange et al., 2013). First, we generated Dox-inducible p57-overexpressing (p57-OE) organoids using a dual PiggyBac transposon system (Figure 4A). p57 expression was monitored by mCherry expression linked to *p57* cDNA via an internal ribosome entry site (IRES) sequence (Figure 4A). 3 days after starting Dox treatment, mCherry signal was visible together with p57 expression, and Ki67 was suppressed (Figure 4B). Importantly, the Dox-induced p57 expression is reversible simply by removing Dox from the culture medium. During 1 week of Dox treatment, organoid growth was suppressed compared with control (Figure S4A). After Dox withdrawal, the growth arrest was released again, indicating that p57 overexpression leads to cell cycle arrest in the organoids as expected (Figure S4A). This effect is conserved in the gastrointestinal tract, as we also observed it in p57-OE organoids from the intestine (Figure S4B), where stem cells are mostly proliferative *in vivo*. We also generated Dox-inducible lines for the other two CIP/KIP family Cdk inhibitors, p21 and p27, and for the other cell cycle inhibitors $p16^{INK4A}$ and $p19^{ARF}$. Overexpressing p21 and p27 in gastric corpus organoids showed a similar effect to the overexpression of p57 but with a significant loss of organoids when the growth arrest was released (Figures S4C and S4D). In contrast, the overexpression of p16 or p19 led to complete differentiation or cell death, respectively (Figures S4E and S4F). These data indicate that p57 induces reversible growth arrest in gastric stem cells with the least harm to stemness among all the tested cell cycle inhibitors.

Next, we wondered whether p57 can induce long-term growth arrest, as seen in RSCs *in vivo*, mimicking the homeostatic chief

(F) UMAP plots for control (light blue) and injury (red) cells.
(G) UMAP plots of *Gif* expression in control (left) and injury (right).
(H) Dot plot for expression of marker genes of each cell type in Gif^{high} and Gif^{low} cell populations.
(I) Dot plot for expression of marker genes of proliferation or cell cycle in Gif^{high} and Gif^{low} cell populations.
(J) Projection of Gif^{high} and Gif^{low} cells on the UMAP plot for Pgc+ scRNA-seq. The cells in control and injury are denoted as light blue and red, respectively. Brown-dotted circles indicate the injury-responsive chief cell population.
(K) UMAP plots of *p57* and *Ki67* expressions in control (left) and injury (right).
See also Figure S3.

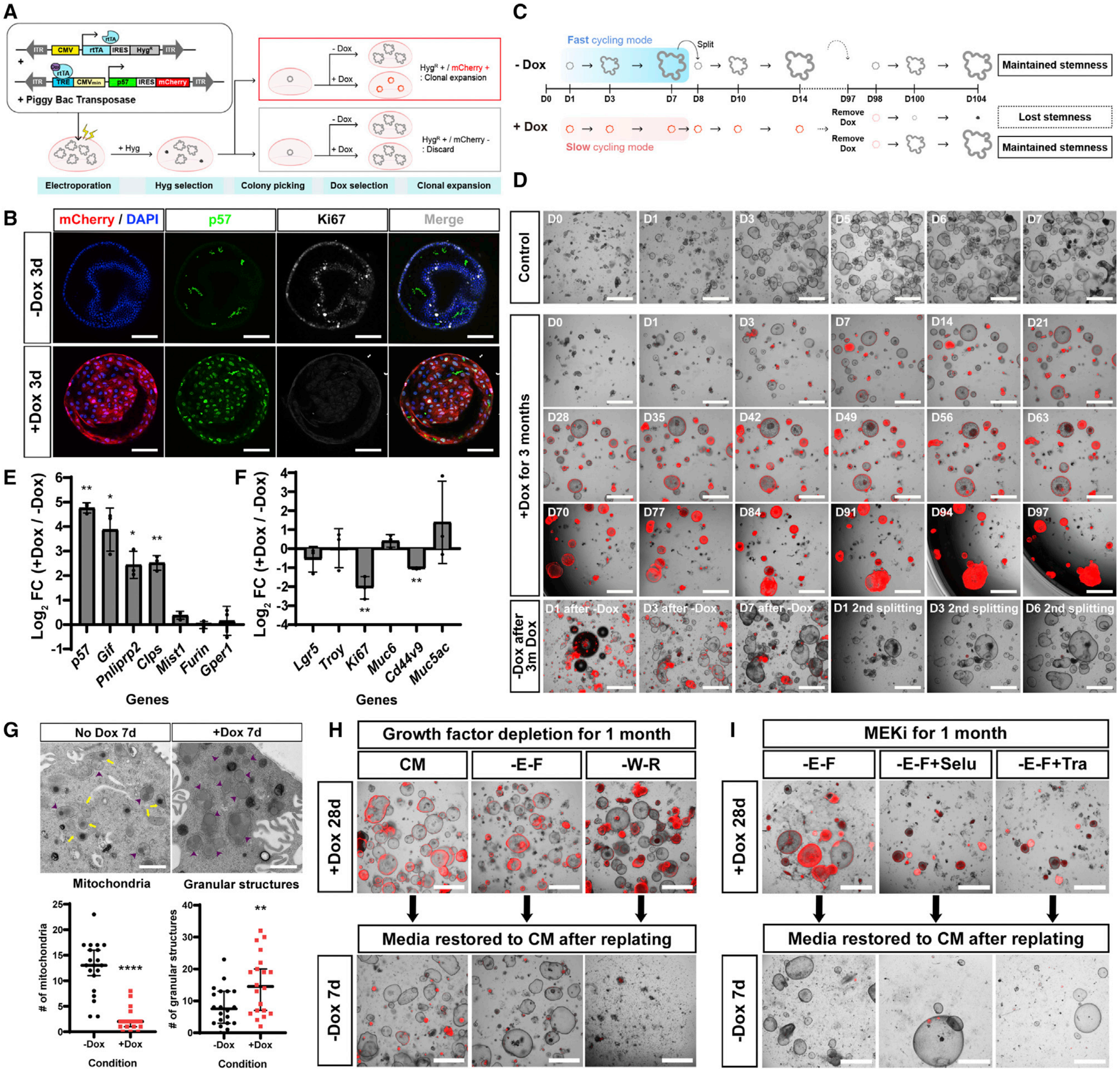

Figure 4. p57 overexpression in gastric organoids triggers long-term growth arrest and enforces a secretory phenotype

(A) Scheme of electroporation generating Dox-inducible p57-OE gastric organoids.

(B) Immunolabeling with p57 and Ki67 antibodies in Dox-inducible p57-OE organoids without (−Dox 3d) or with Dox treatment (+Dox 3d). Scale bars, 100 μm.

(C) Experimental scheme of long-term Dox treatment. Stemness maintenance can be assessed by checking organoid regrowth following Dox withdrawal after long-term Dox treatment.

(D) Induction of long-term growth arrest for 3 months in gastric organoids by p57 expression. Merged images of brightfield and mCherry. The orientation of the organoids changed after D70 as the Matrigel was detached. Organoid regrowth was monitored until the second passage after Dox withdrawal. Several bubbles generated during seeding are visible in “D1 after −Dox.” Scale bars, 1 mm.

(E and F) qRT-PCR of chief cell markers (E) and other markers (F) showing $\log_2$-fold change ($\log_2$ FC) of gene expression in p57-OE organoids (+Dox) compared with no Dox control after 2 weeks of Dox treatment. *Tbp* was used as a reference gene, and the data were generated from three biological replicates. Statistical significances were determined by unpaired multiple t test. *$p < 0.05$, **$p < 0.01$.

(G) Upper images: TEM images of the Dox-inducible p57-OE organoids cultured with (+Dox) or without (−Dox) Dox treatment for 1 week. Yellow arrows indicate mitochondria and purple arrowheads indicate secretory granules. Scale bars, 1 μm. Lower graphs: quantification of the number of mitochondria and granules. Twenty cells of each condition were analyzed. Lines indicate median with 95% confidence interval. Statistical significances were determined by unpaired Welch’s t test. **$p < 0.01$, ****$p < 0.0001$.

(legend continued on next page)

cells in the stomach corpus glands. We treated corpus organoids with Dox for more than 3 months (Figure 4C), which completely suppressed their growth (Figure 4D). Interestingly, upon Dox withdrawal, organoids readily regrew without showing any defects (Figure 4D). This suggests that p57 overexpression induces a RSC-like state in gastric organoids without harming stem cell maintenance. Consequently, even long-term (3 months) growth arrest did not compromise the stem cell program.

To examine whether p57 induces not only growth arrest but also other characteristics of the RSC state, we first checked for changes in the transcription levels of several marker genes using quantitative real-time PCR (qRT-PCR). We found that expression levels of the chief cell markers specifically related to enzymes secreted by chief cells (*Gif*, *Pnliprp2*, and *Clps*) were upregulated after p57 overexpression in gastric organoids (Figure 4E), a sign of chief cell maturation with secretory function. Expression of other chief cell markers like *Mist1*, *Furin*, and *Gper1* was relatively high (Ct value was 28 for *Mist1*, 27 for *Furin*, and 31 for *Gper1* when the Ct of reference gene (*Tbp*) was 29) and constant in both conditions (Figure 4E). Consistently, expression levels of the proliferation marker *Ki67* and the SPEM marker *Cd44v9* were significantly decreased, showing that p57 overexpression makes stem cells less proliferative and less SPEM-like (Figure 4F).

We also compared the ultrastructure of cells from p57-OE organoids with or without Dox by transmission electron microscopy (TEM). In actively cycling organoids, we could easily find mitochondria (yellow arrows), secretory granules (purple arrowheads), and autophagic vesicles (Figure 4G). In p57-OE organoids, however, cells contained very few mitochondria, indicating that they have low energy requirements due to their resting state. In contrast, the number of secretory granules was increased in p57-OE organoids (Figure 4G). In addition, we also found altered niche requirements of p57-OE organoids under their RSC-like state. When p57-OE organoids were cultured without either MAPK signaling activators (EGF and FGF10) or Wnt signaling activators (Wnt3a and Rspo1) for 4 weeks under Dox, all arrested organoids remained viable (Figure 4H). However, after Dox-withdrawal and addition of complete medium, organoids showed rapid regrowth when previously cultured in EGF- and FGF-deficient media but not in Wnt3A- and Rspo1-deficient media (Figure 4H). This was not due to autonomous MAPK pathway activation in p57-OE organoids, as we could see regrowth of organoids after medium restoration, even when they were treated with the MEK inhibitors selumetinib or trametinib in addition to EGF and FGF depletion (Figure 4I). Thus, unlike the normal expanding gastric organoids that require all the niche components contained in gastric organoid medium, i.e., Wnt3A, Rspo1, EGF, FGF10, Noggin, and gastrin (WREFNG) (Barker et al., 2010; Stange et al., 2013), p57-OE organoids do not show any dependence on EGF and FGF. We conclude that p57 expression induces cell cycle arrest of stem and progenitor cells of gastric corpus organoids, with altered niche requirements and mature chief cell characteristics as a RSC.

p57 inhibits the induction of injury-responsive chief cells

To investigate the function of p57 during injury-repair *in vivo*, we crossed the *R26loxpTA-p57* mouse line (Haley et al., 2008) with the stomach-specific *Anxa10-CreERT2* mouse line (Seidlitz et al., 2019). In the *Anxa10-CreER*$^{T2/T2}$; *R26loxpTA-p57*$^{k/k}$ mice, simple HDT administration can induce p57 expression as well as HDT-mediated stomach tissue injury simultaneously (Figure 5A). At 3 days after HDT treatment, the introduced p57 expression was readily observed at the base of *Anxa10-CreER*$^{T2/T2}$; *R26loxpTA-p57*$^{k/k}$ stomach glands, whereas in control mice, p57 expression was dramatically reduced after HDT-mediated tissue injury (Figure 5B). Accordingly, we also found that the damage-induced Ki67 upregulation was seen at the base of the HDT-treated control but not at the HDT-treated p57-expressing epithelium (Figure 5C). Interestingly, these p57-expressing cells also continue to express the chief cell marker Gif (Figure 5D), indicating that these p57-expressing chief cells do not undergo a cell state transition to injury-responsive cells.

Next, we directly assessed whether p57 expression suppresses the induction of the injury-responsive chief cell population. Immunostaining for markers of chief cells (Gif), neck cells (GSII), and proliferating cells (Ki67) showed the induction of double-positive or triple-positive, injury-responsive chief cells in control animals after HDT treatment (Figures 5E–5G), as observed after DMP-777-mediated tissue injury (Figures 2H–2J). However, in the p57-expressing stomach glands, the induction of injury-responsive chief cells was dramatically reduced (Figures 5E–5G). We could also confirm the reduction of the injury response in p57-expressing stomach glands using Cd44v9 and Mist1 staining (Figure S5A). In conclusion, these data show that enforced expression of p57 inhibits the transition of gastric chief cells to injury-responsive chief cells after damage *in vivo*.

To elucidate if p57 knockout also has a phenotype, we first generated a conditional knockout (cKO) allele of *p57* by flanking exon 2 with two loxP sequences. We then generated *Anxa10-CreER*$^{T2/+}$; *p57*$^{loxP/loxP}$ mice and compared their response to HDT with that of *Anxa10-CreER*$^{T2/+}$ control mice. At 14 dpi, when the acute injury has subsided, p57 cKO epithelium showed a dramatic reduction of p57 staining and absence of nuclear localization (Figure S5B). Interestingly, we found persisting injury-responsive chief cells with Gif/Ki67 double-positive or Gif/GSII/Ki67 triple-positive staining (Figure S5C), a sign of a prolonged injury response. We also observed that p57 KO organoids exhibited faster growth than wild-type organoids (Figure S5D). Lastly, we noticed that the decrease of p57 expression observed in wild-type mice after damage occurs several hours

(H) Niche requirements of the p57-OE organoids. p57-OE organoids induced by pretreatment with Dox for 1 week were cultured in each condition together with Dox for 1 month. Organoid growth was examined in complete medium without Dox after replating. CM, complete medium; -E-F, WRNG medium; -W-R, EFNG medium. Scale bars, 1 mm.

(I) p57-OE organoids induced by Dox treatment were cultured in each condition for 1 month. Organoid growth was examined in complete medium without Dox after replating. -E-F, WRNG medium; -E-F+Selu, WRNG medium with selumetinib (100 nM); -E-F+Tra, WRNG medium with trametinib (1 nM). Scale bars, 1 mm. See also Figure S4.

CellPress
OPEN ACCESS

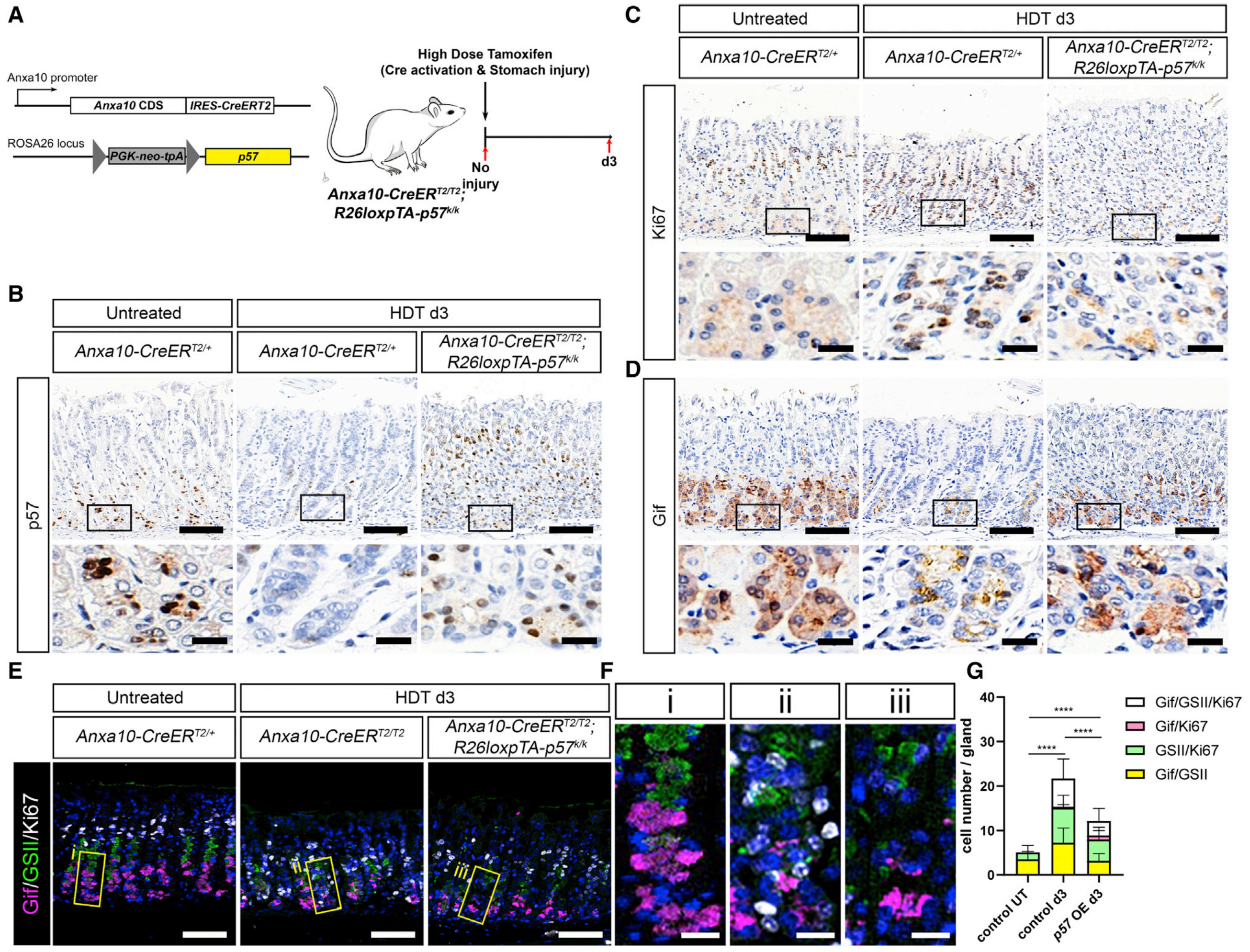

Figure 5. p57 overexpression in stomach epithelium prevents activation of chief cells after injury

(A) Scheme of the *in vivo* experiment.

(B–D) (B) p57, (C) Ki67, and (D) Gif staining in control epithelium without injury (left), control at 3 dpi with HDT (middle), and p57-OE epithelium at 3 dpi with HDT (right). Rectangles indicate insets showing base region. Scale bars, 100 μm (upper figures) and 20 μm (lower figures).

(E) Triple staining with markers for chief cells (Gif, magenta), neck cells (GSII, green), and proliferating cells (Ki67, white) in the conditions as outlined above. Scale bars, 100 μm.

(F) Insets of figure (E). Scale bars, 20 μm.

(G) Quantification of injury-responsive cells ($Gif^+/GS\text{-}II^+$, $Gif^+/Ki67^+$, $GS\text{-}II^+/Ki67^+$, and $Gif^+/GS\text{-}II^+/Ki67^+$). Control UT, *Anxa10-CreERT*$^{2/+}$ untreated; control d3, *Anxa10-CreER*$^{T2/T2}$ HDT d3; *p57* OE d3, *Anxa10-CreER*$^{T2/T2}$; *R26loxpTA-p57*$^{k/k}$ HDT d3. In total, 16–20 glands from 2–3 mice per condition were analyzed. ****$p < 0.0001$ calculated by one-way ANOVA for total injury-responsive cell number.

See also Figure S5.

after the metabolic and molecular changes of chief cell transition that have been reported before (Figure S5E; Miao et al., 2020; Willet et al., 2018). Taken together, we concluded that p57 is a molecular switch that controls the chief cell fate in homeostasis and injury repair.

DISCUSSION

Using transcriptome analysis of Troy+ chief cells during injury repair, here we identified p57 as a candidate molecular switch that regulates the transition between RSC and injury-responsive chief cell. Using scRNA-seq analysis, we found that this transition from p57+ to Ki67+ state happens widely in chief cells upon tissue damage. Characterization of injury-responsive chief cells on a transcriptome level showed that this cell population appears to be a recently reported subpopulation of chief cells that expresses marker genes of neck cells and SPEM (neck+ SPEM+ chief+/−) upon HDT injury (Bockerstett et al., 2020). We carried out a more detailed molecular characterization of injury-responsive chief cells and confirmed their chief cell origin by trajectory analysis as well as Dox-based lineage tracing coupled with scRNA-seq. We then assessed the molecular switch function of p57 *in vitro* and *in vivo*. p57 expression enables chief cells to adopt a RSC state by inducing cell cycle arrest and suppressing the transition to injury-responsive chief cells. p57 expression leads to altered niche requirements with

chief cell maturation characteristics *in vitro*. Our data confirm the previous finding of chief cell plasticity with scRNA-seq and Dox-based lineage tracing. At the same time, we identified the molecular switch that controls chief cell fate in homeostasis and injury repair.

Our observation of chief cell transition to injury-responsive chief cells is in line with a recent publication (Burclaff et al., 2020) that showed chief cell transition to SPEM cells that are double positive for chief and neck cell markers. Unlike the Dox-based Gpr30 (Gper1) lineage tracing study (Hata et al., 2020), our Dox-based Gif lineage tracing confirmed the plasticity of gastric chief cells and their contribution to tissue regeneration upon injury (Caldwell et al., 2021) as observed in the past using tamoxifen-based lineage tracing (Choi et al., 2018; Leushacke et al., 2017; Stange et al., 2013). Gif has been regarded as a specific marker for gastric chief cells (Dieckgraefe et al., 1988), which is confirmed by our scRNA-seq data. The *Gper1* expression domain is similar to that of *Gif*, but *Gper1* seems to be more restricted to the mature chief cell cluster (C11). To explain the discrepancy in lineage tracing results, further studies on chief cell-related subclusters will be needed. We postulate the existence of a common chief-neck progenitor, as we observed intermediate cell types (C/N-1, C/N-2; C4, C1, respectively) during homeostasis in our scRNA-seq data.

p57 is involved in the maintenance of quiescent neural and hematopoietic stem cells (Furutachi et al., 2013; Matsumoto et al., 2011; Zou et al., 2011). It is thought to play a role in the maintenance of the nonproliferative state throughout life (Stampone et al., 2018), whereas p21, a related CKI, is involved in the p53-mediated DNA damage response (He et al., 2005). The gastric chief cells comprise an abundant cell reservoir, which is unique in the gastrointestinal tract, as small intestine and colon utilize differentiating progenitors (Buczacki et al., 2013; Castillo-Azofeifa et al., 2019; van Es et al., 2012; Tetteh et al., 2016; Tomic et al., 2018; Yan et al., 2017; Yu et al., 2018) or developmental progenitor-like cells (Ayyaz et al., 2019) during tissue regeneration upon injury. In our view, in the stomach corpus glands, tissue homeostasis and regeneration are governed by two separate stem cell populations, respectively—constantly proliferating IsthSCs and chief cells as RSCs.

Limitations of the study

In both scRNA-seq analyses, a single time point and a relatively low dose of DMP-777 were used for stomach injury. Although we used different injury methods in the other experiments and showed that the overall reaction is common to all of them, we cannot rule out the possibility that different types of injury could lead to different transcriptomic changes in the activation of chief cells. In addition, from Gif lineage scRNA-seq, we observed that a subset of chief cells successfully entered proliferation upon injury. This might indicate the presence of a subpopulation of the chief cells, but it is also possible that the duration or intensity of the injury determines the number of the fully activated chief cells (Ki67^{+}) during the repair process. Finally, although we found that injury leads to a dramatic change in transcriptome, we do not know whether it also causes epigenetic alterations. Further studies on this transition using a multiomics approach will be needed.

STAR★METHODS

Detailed methods are provided in the online version of this paper and include the following:

- KEY RESOURCES TABLE
- RESOURCE AVAILABILITY
  - Lead contact
  - Materials availability
  - Data and code availability
- EXPERIMENTAL MODEL AND SUBJECT DETAILS
  - Animals
  - Generation of p57 conditional knock-out mice
  - Animal treatments
  - Establishment and culture of gastric corpus organoids from mouse
  - Generation of Dox-inducible OE organoids
- METHOD DETAILS
  - Stomach preparation
  - Immunohistochemistry on paraffin sections
  - Imaging
  - Cell dissociation for bulk RNA-seq and scRNA-seq
  - FACS sorting strategies for bulk RNA-seq and scRNA-seq
  - RNA purification for bulk RNA-seq
  - Library preparation and bulk RNA-seq of Troy^{+} chief cells
  - Generation of single-cell RNA-seq data
  - Immunostaining of p57-OE organoids
  - Quantitative real-time PCR
  - Transmission electron microscopy
- QUANTIFICATION AND STATISTICAL ANALYSIS
  - Statistics
  - Time-course RNA-seq data analysis
  - scRNA-seq data preprocessing
  - scRNA-seq data analysis

SUPPLEMENTAL INFORMATION

Supplemental information can be found online at https://doi.org/10.1016/j.stem.2022.04.001.

ACKNOWLEDGMENTS

pPiggyBac-transposase plasmid was kindly provided by Dr. M. Kinoshita and Prof. G. Jang. We thank the Histology Facility at Vienna BioCenter Core Facilities (VBCF) for immunohistochemistry assistance, N. Drexler at VBC Electron Microscopy Facility for sample preparation and imaging assistance for TEM, the BioOptics facility for automated scanning assistance, and T. Jeong and H. Lee for organoid imaging assistance. We also thank Single Cell Discoveries for their single-cell sequencing services and data analysis. A.P. is supported by core support from the Wellcome Trust and the MRC Cambridge Stem Cell Institute. We are grateful to Dr. J.C. Marioni for help with scRNA-seq analysis, to Dr. R. Zietlow for critical reading of the manuscript, and to S.H. Bae for graphic illustration. This work was supported by a number of grants, fellowships, and studentships: S.H. (HFSP LT000092/2016-L; Basic Science Research Program NRF-2014R1A6A3A01005675), J.M. (DOD CA191242), J.F. (Wellcome Trust), A.P. (Cancer Research UK Programme Grant, A25636; Wellcome Trust Investigator Award, 212253/Z/18/Z), B.D.S. (the Royal Scientific Society E.P. Abraham Research Professorship, RP\R1\180165; Wellcome Trust, 098357/Z/12/Z and 219478/Z/19/Z), E.C. (DOD CA160399 and NIH R37 CA244970 and pilot funding from Vanderbilt

DDRC DK058404 and VICC GI SPORE P50CA236733), B.-K.K. (ERC starting grant, Troy Stem Cells, 639050; the Austrian Academy of Sciences; Human Frontier Science Program; Interpark Bio-Convergence Center Grant Program; Institute for Basic Science, IBS-R021-D1), and J.K.K. (2020R1A2C4001630, 2017M3A9B6073099, and the DGIST R&D program [20-CoE-BT-04] funded by the Ministry of Science and ICT of Korea). This research was funded in part by the Wellcome Trust (203151/Z/16/Z) and the UKRI Medical Research Council (MC_PC_17230). For the purpose of open access, the author has applied a CC BY public copyright license to any Author Accepted Manuscript version arising from this submission.

AUTHOR CONTRIBUTIONS

J.-H.L., S.H., J.M., E.C., B.-K.K., and J.K.K. designed the experiments. J.-H.L., S.H., J.M., B.C., A.-D.B., A.S.B.R., J.P., S.L., S.-H.S.W., J.F., S.P.-C., J.K., M.J., R.S.-B., and Y.A. performed animal experiments, organoid experiments, tissue and organoid imaging, and quantification that were supervised by Y.S.J., A.P., B.D.S., D.E.S., E.C., B.-K.K., and J.K.K. S.K., S.H., H.L., and Y.A. analyzed bulk RNA-seq and single-cell RNA-seq data, which were supervised by Y.S.J., B.-K.K., and J.K.K. J.-H.L., S.K., J.M., E.C., B.-K.K., and J.K.K. wrote the manuscript with support from all the authors.

DECLARATION OF INTERESTS

The authors declare no competing interests.

Received: April 12, 2021
Revised: February 17, 2022
Accepted: April 1, 2022
Published: May 5, 2022

REFERENCES

Alexa, A., and Rahnenfuhrer, J.. topGO: Enrichment Analysis for Gene Ontology. R package version 2.36.0. https://bioconductor.org/packages/topGO/.

Anders, S., Pyl, P.T., and Huber, W. (2015). HTSeq—a Python framework to work with high-throughput sequencing data. Bioinformatics *31*, 166–169.

Andrews, S.. FastQC: a quality control tool for high throughput sequence data. http://www.bioinformatics.babraham.ac.uk/projects/fastqc.

Arnold, K., Sarkar, A., Yram, M.A., Polo, J.M., Bronson, R., SenGupta, S., Seandel, M., Geijsen, N., and Hochedlinger, K. (2011). Sox2+ adult stem and progenitor cells are important for tissue regeneration and survival of mice. Cell Stem Cell *9*, 317–329.

Ayyaz, A., Kumar, S., Sangiorgi, B., Ghoshal, B., Gosio, J., Ouladan, S., Fink, M., Barutcu, S., Trcka, D., Shen, J., et al. (2019). Single-cell transcriptomes of the regenerating intestine reveal a revival stem cell. Nature *569*, 121–125.

Barker, N., Huch, M., Kujala, P., van de Wetering, M., Snippert, H.J., van Es, J.H., Sato, T., Stange, D.E., Begthel, H., van den Born, M., et al. (2010). Lgr5+ve stem cells drive self-renewal in the stomach and build long-lived gastric units *in vitro*. Cell Stem Cell *6*, 25–36.

Bartfeld, S., Bayram, T., van de Wetering, M., Huch, M., Begthel, H., Kujala, P., Vries, R., Peters, P.J., and Clevers, H. (2015). *In vitro* expansion of human gastric epithelial stem cells and their responses to bacterial infection. Gastroenterology *148*, 126–136.e6.

Bergen, V., Lange, M., Peidli, S., Wolf, F.A., and Theis, F.J. (2020). Generalizing RNA velocity to transient cell states through dynamical modeling. Nat. Biotechnol. *38*, 1408–1414.

Bockerstett, K.A., Lewis, S.A., Wolf, K.J., Noto, C.N., Jackson, N.M., Ford, E.L., Ahn, T.H., and Dipaolo, R.J. (2020). Single-cell transcriptional analyses of spasmolytic polypeptide-expressing metaplasia arising from acute drug injury and chronic inflammation in the stomach. Gut *69*, 1027–1038.

Buczacki, S.J.A., Zecchini, H.I., Nicholson, A.M., Russell, R., Vermeulen, L., Kemp, R., and Winton, D.J. (2013). Intestinal label-retaining cells are secretory precursors expressing Lgr5. Nature *495*, 65–69.

Burclaff, J., Willet, S.G., Sáenz, J.B., and Mills, J.C. (2020). Proliferation and differentiation of gastric mucous neck and chief cells during homeostasis and injury-induced metaplasia. Gastroenterology *158*, 598–609.e5.

Busslinger, G.A., Weusten, B.L.A., Bogte, A., Begthel, H., Brosens, L.A.A., and Clevers, H. (2021). Human gastrointestinal epithelia of the esophagus, stomach, and duodenum resolved at single-cell resolution. Cell Rep. *34*, 108819.

Butler, A., Hoffman, P., Smibert, P., Papalexi, E., and Satija, R. (2018). Integrating single-cell transcriptomic data across different conditions, technologies, and species. Nat. Biotechnol. *36*, 411–420.

Caldwell, B., Meyer, A.R., Weis, J.A., Engevik, A.C., and Choi, E. (2021). Chief cell plasticity is the origin of metaplasia following acute injury in the stomach mucosa. Gut. http://dx.doi.org/10.1136/gutjnl-2021-325310. (Accessed 8 September 2021).

Carlson, M. (2019). org.Mm.eg.db: Genome wide annotation for Mouse. R package version 3.8.2. https://bioconductor.org/packages/org.Mm.eg.db/.

Carvalho, B.S., and Irizarry, R.A. (2010). A framework for oligonucleotide microarray preprocessing. Bioinformatics *26*, 2363–2367.

Castillo-Azofeifa, D., Fazio, E.N., Nattiv, R., Good, H.J., Wald, T., Pest, M.A., de Sauvage, F.J., Klein, O.D., and Asfaha, S. (2019). Atoh1+ secretory progenitors possess renewal capacity independent of Lgr5+ cells during colonic regeneration. EMBO J. *38*, e99984.

Cheung, T.H., and Rando, T.A. (2013). Molecular regulation of stem cell quiescence. Nat. Rev. Mol. Cell Biol. *14*, 329–340.

Choi, E., Lantz, T.L., Vlacich, G., Keeley, T.M., Samuelson, L.C., Coffey, R.J., Goldenring, J.R., and Powell, A.E. (2018). Lrig1+ gastric isthmal progenitor cells restore normal gastric lineage cells during damage recovery in adult mouse stomach. Gut *67*, 1595–1605.

Dieckgraefe, B.K., Seetharam, B., and Alpers, D.H. (1988). Developmental regulation of rat intrinsic factor mRNA. Am. J. Physiol. *254*, G913–G919.

Dobin, A., Davis, C.A., Schlesinger, F., Drenkow, J., Zaleski, C., Jha, S., Batut, P., Chaisson, M., and Gingeras, T.R. (2013). STAR: ultrafast universal RNA-seq aligner. Bioinformatics *29*, 15–21.

Durinck, S., Moreau, Y., Kasprzyk, A., Davis, S., De Moor, B., Brazma, A., and Huber, W. (2005). BioMart and Bioconductor: a powerful link between biological databases and microarray data analysis. Bioinformatics *21*, 3439–3440.

Furutachi, S., Matsumoto, A., Nakayama, K.I., and Gotoh, Y. (2013). P57 controls adult neural stem cell quiescence and modulates the pace of lifelong neurogenesis. EMBO J. *32*, 970–981.

Ganger, M.T., Dietz, G.D., and Ewing, S.J. (2017). A common base method for analysis of qPCR data and the application of simple blocking in qPCR experiments. BMC Bioinformatics *18*, 534.

Haley, S.A., Zhao, T., Zou, L., Klysik, J.E., Padbury, J.F., and Kochilas, L.K. (2008). Forced expression of the cell cycle inhibitor p57Kip2 in cardiomyocytes attenuates ischemia-reperfusion injury in the mouse heart. BMC Physiol. *8*, 4.

Han, S., Fink, J., Jörg, D.J., Lee, E., Yum, M.K., Chatzeli, L., Merker, S.R., Josserand, M., Trendafilova, T., Andersson-Rolf, A., et al. (2019). Defining the identity and dynamics of adult gastric isthmus stem cells. Cell Stem Cell *25*, 342–356.e7.

Hashimshony, T., Senderovich, N., Avital, G., Klochendler, A., de Leeuw, Y., Anavy, L., Gennert, D., Li, S., Livak, K.J., Rozenblatt-Rosen, O., et al. (2016). CEL-Seq2: sensitive highly-multiplexed single-cell RNA-seq. Genome Biol. *17*, 77.

Hata, M., Kinoshita, H., Hayakawa, Y., Konishi, M., Tsuboi, M., Oya, Y., Kurokawa, K., Hayata, Y., Nakagawa, H., Tateishi, K., et al. (2020). GPR30-expressing gastric chief cells do not dedifferentiate but are eliminated via PDK-dependent cell competition During development of metaplasia. Gastroenterology *158*, 1650–1666.e15.

He, G., Siddik, Z.H., Huang, Z., Wang, R., Koomen, J., Kobayashi, R., Khokhar, A.R., and Kuang, J. (2005). Induction of p21 by p53 following DNA damage inhibits both Cdk4 and Cdk2 activities. Oncogene *24*, 2929–2943.

Hennig, C. (2020). fpc: flexible procedures for clustering. https://www.unibo.it/sitoweb/christian.hennig/en/.

Huh, W.J., Khurana, S.S., Geahlen, J.H., Kohli, K., Waller, R.A., and Mills, J.C. (2012). Tamoxifen induces rapid, reversible atrophy, and metaplasia in mouse stomach. Gastroenterology *142*, 21–24.e7.

Janda, C.Y., Dang, L.T., You, C., Chang, J., de Lau, W., Zhong, Z.A., Yan, K.S., Marecic, O., Siepe, D., Li, X., et al. (2017). Surrogate Wnt agonists that phenocopy canonical Wnt and β-catenin signalling. Nature *545*, 234–237.

La Manno, G., Soldatov, R., Zeisel, A., Braun, E., Hochgerner, H., Petukhov, V., Lidschreiber, K., Kastriti, M.E., Lönnerberg, P., Furlan, A., et al. (2018). RNA velocity of single cells. Nature *560*, 494–498.

Leushacke, M., Tan, S.H., Wong, A., Swathi, Y., Hajamohideen, A., Tan, L.T., Goh, J., Wong, E., Denil, S.L.I.J., Murakami, K., and Barker, N. (2017). Lgr5-expressing chief cells drive epithelial regeneration and cancer in the oxyntic stomach. Nat. Cell Biol. *19*, 774–786.

Li, H., Handsaker, B., Wysoker, A., Fennell, T., Ruan, J., Homer, N., Marth, G., Abecasis, G., and Durbin, R.; 1000 Genome Project Data Processing Subgroup (2009). The Sequence Alignment/Map format and SAMtools. Bioinformatics *25*, 2078–2079.

Liberzon, A., Subramanian, A., Pinchback, R., Thorvaldsdóttir, H., Tamayo, P., and Mesirov, J.P. (2011). Molecular signatures database (MSigDB) 3.0. Bioinformatics *27*, 1739–1740.

Liu, L., Michowski, W., Kolodziejczyk, A., and Sicinski, P. (2019). The cell cycle in stem cell proliferation, pluripotency and differentiation. Nat. Cell Biol. *21*, 1060–1067.

Love, M.I., Huber, W., and Anders, S. (2014). Moderated estimation of fold change and dispersion for RNA-seq data with DESeq2. Genome Biol. *15*, 550.

Lun, A.T.L., McCarthy, D.J., and Marioni, J.C. (2016). A step-by-step workflow for low-level analysis of single-cell RNA-seq data with Bioconductor. F1000Res *5*, 2122.

Mahe, M.M., Aihara, E., Schumacher, M.A., Zavros, Y., Montrose, M.H., Helmrath, M.A., Sato, T., and Shroyer, N.F. (2013). Establishment of gastrointestinal epithelial organoids. Curr. Protoc. Mouse Biol. *3*, 217–240.

Matsumoto, A., Takeishi, S., Kanie, T., Susaki, E., Onoyama, I., Tateishi, Y., Nakayama, K., and Nakayama, K.I. (2011). P57 Is required for quiescence and maintenance of adult hematopoietic stem cells. Cell Stem Cell *9*, 262–271.

McCarthy, D.J., Campbell, K.R., Lun, A.T.L., and Wills, Q.F. (2017). Scater: pre-processing, quality control, normalization and visualization of single-cell RNA-seq data in R. Bioinformatics *33*, 1179–1186.

Menheniott, T.R., Peterson, A.J., O'Connor, L., Lee, K.S., Kalantzis, A., Kondova, I., Bontrop, R.E., Bell, K.M., and Giraud, A.S. (2010). A novel Gastrokine, Gkn3, marks gastric atrophy and shows evidence of adaptive gene loss in humans. Gastroenterology *138*, 1823–1835.

Merenda, A., Andersson-Rolf, A., Mustata, R.C., Li, T., Kim, H., and Koo, B.K. (2017). A protocol for multiple gene knockout in mouse small intestinal organoids using a CRISPR-concatemer. J. Vis. Exp. *125*, e55916.

Miao, Z.F., Lewis, M.A., Cho, C.J., Adkins-Threats, M., Park, D., Brown, J.W., Sun, J.X., Burclaff, J.R., Kennedy, S., Lu, J., et al. (2020). A dedicated evolutionarily conserved molecular network licenses differentiated cells to return to the cell cycle. Dev. Cell *55*, 178–194.e7.

Mootha, V.K., Lindgren, C.M., Eriksson, K.-F., Subramanian, A., Sihag, S., Lehar, J., Puigserver, P., Carlsson, E., Ridderstråle, M., Laurila, E., et al. (2003). PGC-1α-responsive genes involved in oxidative phosphorylation are coordinately downregulated in human diabetes. Nat. Genet. *34*, 267–273.

Mouselimis, L. (2021). KernelKnn: kernel k nearest Neighbors. https://github.com/mlampros/KernelKnn.

Muñoz, J., Stange, D.E., Schepers, A.G., Van De Wetering, M., Koo, B.K., Itzkovitz, S., Volckmann, R., Kung, K.S., Koster, J., Radulescu, S., et al. (2012). The Lgr5 intestinal stem cell signature: robust expression of proposed quiescent ′ +4′ cell markers. EMBO J. *31*, 3079–3091.

Muraro, M.J., Dharmadhikari, G., Grün, D., Groen, N., Dielen, T., Jansen, E., van Gurp, L., Engelse, M.A., Carlotti, F., de Koning, E.J.P., and van Oudenaarden, A. (2016). A single-cell transcriptome atlas of the human pancreas. Cell Syst. *3*, 385–394.e3.

Nam, K.T., Lee, H.J., Sousa, J.F., Weis, V.G., O'Neal, R.L., Finke, P.E., Romero-Gallo, J., Shi, G., Mills, J.C., Peek, R.M., et al. (2010). Mature chief cells are cryptic progenitors for metaplasia in the stomach. Gastroenterology *139*, 2028–2037.e9.

Nomura, S., Yamaguchi, H., Ogawa, M., Wang, T.C., Lee, J.R., and Goldenring, J.R. (2005). Alterations in gastric mucosal lineages induced by acute oxyntic atrophy in wild-type and gastrin-deficient mice. Am. J. Physiol. Gastrointest. Liver Physiol. *288*, G362–G375.

Nozaki, K., Ogawa, M., Williams, J.A., Lafleur, B.J., Ng, V., Drapkin, R.I., Mills, J.C., Konieczny, S.F., Nomura, S., and Goldenring, J.R. (2008). A molecular signature of gastric metaplasia arising in response to acute parietal cell loss. Gastroenterology *134*, 511–522.

Parekh, S., Ziegenhain, C., Vieth, B., Enard, W., and Hellmann, I. (2018). zUMIs—a fast and flexible pipeline to process RNA sequencing data with UMIs. GigaScience *7*, giy059.

Picelli, S., Faridani, O.R., Björklund, A.K., Winberg, G., Sagasser, S., and Sandberg, R. (2014). Full-length RNA-seq from single cells using Smart-seq2. Nat. Protoc. *9*, 171–181.

Ritchie, M.E., Phipson, B., Wu, D., Hu, Y., Law, C.W., Shi, W., and Smyth, G.K. (2015). limma powers differential expression analyses for RNA-sequencing and microarray studies. Nucleic Acids Res. *43*, e47.

Seidlitz, T., Chen, Y.T., Uhlemann, H., Schölch, S., Kochall, S., Merker, S.R., Klimova, A., Hennig, A., Schweitzer, C., Pape, K., et al. (2019). Mouse models of human gastric cancer subtypes With stomach-specific CreERT2-mediated pathway alterations. Gastroenterology *157*, 1599–1614.e2.

Setty, M., Kiseliovas, V., Levine, J., Gayoso, A., Mazutis, L., and Pe'er, D. (2019). Characterization of cell fate probabilities in single-cell data with Palantir. Nat. Biotechnol. *37*, 451–460.

Stampone, E., Caldarelli, I., Zullo, A., Bencivenga, D., Mancini, F.P., Della Ragione, F., and Borriello, A. (2018). Genetic and epigenetic control of CDKN1C expression: importance in cell commitment and differentiation, tissue homeostasis and human diseases. Int. J. Mol. Sci. *19*, 1–24.

Stange, D.E., Koo, B.K., Huch, M., Sibbel, G., Basak, O., Lyubimova, A., Kujala, P., Bartfeld, S., Koster, J., Geahlen, J.H., et al. (2013). Differentiated Troy+ chief cells act as reserve stem cells to generate all lineages of the stomach epithelium. Cell *155*, 357–368.

Street, K., Risso, D., Fletcher, R.B., Das, D., Ngai, J., Yosef, N., Purdom, E., and Dudoit, S. (2018). Slingshot: cell lineage and pseudotime inference for single-cell transcriptomics. BMC Genomics *19*, 477.

Stuart, T., Butler, A., Hoffman, P., Hafemeister, C., Papalexi, E., Mauck, W.M., Hao, Y., Stoeckius, M., Smibert, P., and Satija, R. (2019). Comprehensive integration of single-cell data. Cell *177*, 1888–1902.e21.

Subramanian, A., Tamayo, P., Mootha, V.K., Mukherjee, S., Ebert, B.L., Gillette, M.A., Paulovich, A., Pomeroy, S.L., Golub, T.R., Lander, E.S., and Mesirov, J.P. (2005). Gene set enrichment analysis: a knowledge-based approach for interpreting genome-wide expression profiles. Proc. Natl. Acad. Sci. USA *102*, 15545–15550.

Tetteh, P.W., Basak, O., Farin, H.F., Wiebrands, K., Kretzschmar, K., Begthel, H., van den Born, M., Korving, J., de Sauvage, F., van Es, J.H., et al. (2016). Replacement of lost Lgr5-positive stem cells through plasticity of their enterocyte-lineage daughters. Cell Stem Cell *18*, 203–213.

Tomic, G., Morrissey, E., Kozar, S., Ben-Moshe, S., Hoyle, A., Azzarelli, R., Kemp, R., Chilamakuri, C.S.R., Itzkovitz, S., Philpott, A., and Winton, D.J. (2018). Phospho-regulation of ATOH1 is required for plasticity of secretory progenitors and tissue regeneration. Cell Stem Cell *23*, 436–443.e7.

van den Brink, S.C., Sage, F., Vértesy, Á., Spanjaard, B., Peterson-Maduro, J., Baron, C.S., Robin, C., and van Oudenaarden, A. (2017). Single-cell sequencing reveals dissociation-induced gene expression in tissue subpopulations. Nat. Methods *14*, 935–936.

van Es, J.H., Sato, T., van de Wetering, M., Lyubimova, A., Yee Nee, A.N., Gregorieff, A., Sasaki, N., Zeinstra, L., van den Born, M., Korving, J., et al. (2012). Dll1+ secretory progenitor cells revert to stem cells upon crypt damage. Nat. Cell Biol. *14*, 1099–1104.

Willet, S.G., Lewis, M.A., Miao, Z., Liu, D., Radyk, M.D., Cunningham, R.L., Burclaff, J., Sibbel, G., Lo, H.G., Blanc, V., et al. (2018). Regenerative

CellPress
OPEN ACCESS

proliferation of differentiated cells by mTORC 1-dependent paligenosis. EMBO J. *37*, 1–18.

Wolf, F.A., Angerer, P., and Theis, F.J. (2018). SCANPY: large-scale single-cell gene expression data analysis. Genome Biol. *19*, 15.

Wu, M., and Gu, L.. TCseq: Time course sequencing data analysis. R package version 1.10.0. https://bioconductor.org/packages/TCseq/.

Yan, K.S., Gevaert, O., Zheng, G.X.Y., Anchang, B., Probert, C.S., Larkin, K.A., Davies, P.S., Cheng, Z.F., Kaddis, J.S., Han, A., et al. (2017). Intestinal enteroendocrine lineage cells possess homeostatic and injury-inducible stem cell activity. Cell Stem Cell *21*, 78–90.e6.

Yu, S., Tong, K., Zhao, Y., Balasubramanian, I., Yap, G.S., Ferraris, R.P., Bonder, E.M., Verzi, M.P., and Gao, N. (2018). Paneth cell multipotency induced by Notch activation following Injury. Cell Stem Cell *23*, 46–59.e5.

Zou, P., Yoshihara, H., Hosokawa, K., Tai, I., Shinmyozu, K., Tsukahara, F., Maru, Y., Nakayama, K., Nakayama, K.I., and Suda, T. (2011). P57Kip2and p27Kip1cooperate to maintain hematopoietic stem cell quiescence through interactions with Hsc70. Cell Stem Cell *9*, 247–261.

STAR★METHODS

KEY RESOURCES TABLE

REAGENT or RESOURCE	SOURCE	IDENTIFIER
Antibodies		
ATP4B Polyclonal Antibody, Alexa Fluor 555 Conjugated	Bioss	Cat# bs-2433R-A555; RRID: AB_2909527
mouse anti-H,K-ATPase α subunit	MBL	Cat# D031-3; RRID: AB_590576
rabbit anti-Ki67	A. Menarini	Cat# MP-325-CRM1
rabbit anti-Ki67	Abcam	Cat# ab16667; RRID: AB_302459
rabbit anti-Gif	Sigma-Aldrich	Cat# HPA040774; RRID: AB_10795626
rabbit anti-p57	Abcam	Cat# ab75974; RRID: AB_1310535
Rabbit anti-mouse link	Abcam	Cat# ab133469; RRID: AB_2910607
rat anti-Ki67 (SolA15), eBioscience™	Invitrogen	Cat# 14-5698-82; RRID: AB_10854564
rat anti-Ki67	Biolegend	Cat# 652402; RRID: AB_11204254
goat anti-GFP	Novus	Cat# NB100-1770ss; RRID: AB_10128178
rabbit anti-Mist1/bHLHa15	Cell Signaling	Cat# 14896S; RRID: AB_2798639
mouse anti-p120 catenin	BD Biosciences	Cat# 610133; RRID: AB_397536
rat anti-CD44v9	Cosmo Bio	Cat# CAC-LKG-M002; RRID: AB_2910608
rabbit anti-pS6 240/244	Cell Signaling	Cat# 5364; RRID: AB_10694233
rat anti-Lamp1	DSHB	Cat# 1D4B; RRID: AB_2134500
FITC-conjugated-UEAI-Lectin	Sigma	Cat# L9006
Alexa 647-conjugated GS-II Lectin	Invitrogen	Cat# L32451
Alexa 488-conjugated GS-II Lectin	Invitrogen	Cat# L21415
donkey anti-rat IgG (H+L) Cross-adsorbed DyLight 650	Invitrogen	Cat# SA5-10029; RRID: AB_2556609
donkey anti-rabbit IgG H&L, Alexa Fluor 488	Abcam	Cat# ab150073; RRID: AB_2636877
donkey anti-goat IgG (H+L) Highly Cross-Adsorbed Secondary Antibody, Alexa Fluor 488	ThermoFisher	Cat# A11055; RRID: AB_2534102
donkey anti-rabbit IgG (H+L) Highly Cross-Adsorbed Secondary Antibody, Alexa Fluor 546	ThermoFisher	Cat# A10040; RRID: AB_2534016
donkey anti-mouse IgG (H+L) Highly Cross-Adsorbed Secondary Antibody, Alexa Fluor 790	ThermoFisher	Cat# A11371; RRID: AB_2534144
Alexa Fluor 647 anti-mouse/human CD324 (E-Cadherin) Antibody	Biolegend	Cat# 147308; RRID: AB_2563955
Chemicals, peptides, and recombinant proteins		
DMP-777	Matrix Scientific	Cat# 96053
DMP-777	MedChemExpress	Cat# HY-75957
L-635	Vanderbilt ChemicalSynthesis Core	N/A
Doxycycline Hyclate	Sigma-Aldrich	Cat# D9891
tamoxifen	Sigma-Aldrich	Cat# T5648
Advanced DMEM/F-12	Thermofisher Scientific	Cat# 12634028
GlutaMAX™ Supplement	Gibco	Cat# 35050061
HEPES (1M)	Gibco	Cat# 15630056
penicillin/ streptomycin	Thermo Fisher Scientific	Cat# 15140122
Wnt3A conditioned medium	N/A	N/A
recombinant Wnt-surrogate	U-protein express	Cat# N001
Rspo1 conditioned medium	N/A	N/A
B27 supplement	Gibco	Cat# 17504044
N-acetyl-N.-cysteine	Sigma-Aldrich	Cat# A9165
mouse EGF	Gibco	Cat# PMG8043

(Continued on next page)

Continued

REAGENT or RESOURCE	SOURCE	IDENTIFIER
mouse Noggin	Peprotech	Cat# 250-38
recombinant hNoggin	Interpark	N/A
hFgf10	Peprotech	Cat# AF-100-26
Gastrin	Sigma-Aldrich	Cat# G9145
Y-27632 (ROCK-inhibitor)	Adooq Bioscience	Cat# A11001-50
Matrigel, BasementMembrane Matrix, Growth Factor Reduced (GFR), Phenol Red-free	Corning	Cat# BDL356231
Hygromycin B Gold	InvivoGen	Cat# ant-hg-1
Selumetinib	Med Chem Express	Cat# HY-50706
Trametinib	Med Chem Express	Cat# HY-10999
paraformaldehyde	Sigma-Aldrich	Cat# P6148
10% neutral bufferedformalin	Sigma-Aldrich	Cat# ht501128
Dispase II	Thermo Fisher Scientific	Cat# 17105041
pancreatin	Sigma	Cat# P3292
FBS	Sigma-Aldrich	Cat# F7524
DNAse I (lipophilized)	Roche	Cat# 4536282001
Gentle Cell Dissociation Reagent	Stemcell Technologies	Cat# 7174
DMEM high glucose, HEPES, no phenol red	Thermofisher Scientific	Cat# 21063029
SuperVision 2 HRP Single Species: 2-step polymer system, peroxidase conjugated, rabbit	DCS	Cat# PD000P OL-K
mounting solution	Vectashiled	Cat# VECH-1900-10
**Deposited data**		
bulk RNA-seq data from the Troy-eGFP KI model	EMBL-EBI	ArrayExpress: E-MTAB-10373
single-cell RNA-seq data from Pgc-DsRed KI model	EMBL-EBI	ArrayExpress: E-MTAB-10371
single-cell RNA-seq data of Gif lineage tracing model	EMBL-EBI	ArrayExpress: E-MTAB-11587
**Experimental models: Organisms/strains**		
C57BL/6J	Jackson Laboratory	Cat# 000664; RRID:IMSR_JAX:000664
Troy-eGFP-IRES-CreERT2	Stange et al., 2013	MGI ID:5613002
Pgc-IRES-DTR-T2A-dsRed	Han et al., 2019	MGI ID:6456666
Anxa10-CreERT2	Seidlitz et al., 2019	N/A
R26loxpTA-p57	Jackson Laboratory	Cat# 022516; RRID:IMSR_JAX:022516
TetO-Cre	Jackson Laboratory	Cat# 006224; RRID:IMSR_JAX:006224
Gt(ROSA)26Sor$^{tm1(CAG-tdTomato*,-EGFP*)Ees}$*/J*	Jackson Laboratory	Cat# 023537; RRID:IMSR_JAX:023537
Gif-rtTA	Caldwell et al., 2021	N/A
p57$^{loxP/loxP}$	This paper	N/A
**Oligonucleotides**		
Primers for qRT-PCR, see Table S3	This paper	N/A
**Recombinant DNA**		
pPiggyBac transposase	Laboratory of Prof. G. Jang	N/A
pPB-CAG-rtTA-IRES-Hygro (CMV-rtTA-HygR)	Addgene	Cat# 102423; RRID:Addgene_102423
pPB-CMVmin-TRE-p57-IRES-mCherry	This paper	N/A
pPB-CMVmin-TRE-p21-IRES-mCherry	This paper	N/A
pPB-CMVmin-TRE-p27-IRES-mCherry	This paper	N/A
pPB-CMVmin-TRE-p16-IRES-mCherry	This paper	N/A
pPB-CMVmin-TRE-p19^{ARF}-IRES-mCherry	This paper	N/A
**Software and algorithms**		
Image J	Schneider et al., 2012	http://imagej.nih.gov/ij
GraphPad Prism 8	Graphpad software	https://www.graphpad.com/scientific-software/prism/

(*Continued on next page*)

Continued

REAGENT or RESOURCE	SOURCE	IDENTIFIER
CaseViewer (v2.4.0)	3DHISTECH Ltd.	https://www.3dhistech.com/research/software/software-downloads/
FastQC	Andrews, 2010	https://www.bioinformatics.babraham.ac.uk/projects/fastqc/
STAR (v2.5.2b)	Dobin et al., 2013	https://github.com/alexdobin/STAR
SAMtools (v1.4)	Li et al., 2009	http://www.htslib.org/
HTseq (v0.7.2)	Anders et al., 2015	https://htseq.readthedocs.io/en/master/
TCseq (v1.10.0)	Wu and Gu, 2020	https://www.bioconductor.org/packages/release/bioc/html/TCseq.html
fpc (v.2.2-8)	Hennig, 2020	https://cran.r-project.org/web/packages/fpc/index.html
topGO (v2.36.0)	Alexa and Rahnenfuhrer, 2019	https://www.bioconductor.org/packages/release/bioc/html/topGO.html
GSEA (v4.1.0)	Mootha et al., 2003; Subramanian et al., 2005	https://www.gsea-msigdb.org/gsea/index.jsp
DEseq2 (v1.26.0)	Love et al., 2014	https://www.bioconductor.org/packages/release/bioc/html/DESeq2.html
biomaRt (v2.46.0)	Durinck et al., 2005	https://www.bioconductor.org/packages/release/bioc/html/biomaRt.html
oligo (v1.54.1)	Carvalho and Irizarry, 2010	https://www.bioconductor.org/packages/release/bioc/html/oligo.html
limma (v3.46.0)	Ritchie et al., 2015	https://www.bioconductor.org/packages/release/bioc/html/limma.html
scater (v1.14.0)	McCarthy et al., 2017	https://www.bioconductor.org/packages/release/bioc/html/scater.html
scran (v1.14.6)	Lun et al., 2016	https://www.bioconductor.org/packages/release/bioc/html/scran.html
Seurat (v3.1.4)	Butler et al., 2018; Stuart et al., 2019	https://github.com/satijalab/seurat
scVelo (v0.2.1)	Bergen et al., 2020	https://github.com/theislab/scvelo
velocyto (v0.17.17)	La Manno et al., 2018	https://github.com/velocyto-team/velocyto.R
Palantir (v0.2.6)	Setty et al., 2019	https://github.com/dpeerlab/Palantir
PAGA	Wolf et al., 2019	https://github.com/theislab/paga
scanpy (v1.5.1)	Wolf et al., 2018	https://github.com/theislab/scanpy
KernelKnn (v1.1.2)	Mouselimis, 2021	https://github.com/mlampros/KernelKnn
slingshot (v1.4.0)	Street et al., 2018	https://github.com/kstreet13/slingshot
Datasets/codes for single-cell RNA-seq analysis	This paper	https://github.com/scg-dgist/gastric-chief-cell
Other		
µ-Slide 8 Well	ibidi	Cat# 80826
48 well plate	Corning	Cat# CLS3548
Cell culture plate 96 well	Eppendorf	Cat# 30730119
SuperScript III reverse transcriptase	Invitrogen	Cat# 18080044
GoTaq qPCR master mix	Promega	Cat# A6001
KAPA HiFi HotStart ReadyMix	Roche	Cat# 7958927001
Nextera XT DNA sample preparation kit	Illumina	Cat# FC-131-1096
Arcturus Pico Pure RNA Isolation Kit	Applied Biosystems	Cat# KIT0204

RESOURCE AVAILABILITY

Lead contact

Further information and requests for resources and reagents should be directed to, and will be fulfilled by, the lead contact, Dr. Bon-Kyoung Koo (koobk@ibs.re.kr).

CellPress
OPEN ACCESS

Materials availability

All unique/stable reagents generated in this study will be freely available from the lead contact to academic researchers with a completed Materials Transfer Agreement.

Data and code availability

The bulk RNA-seq data from the Troy-eGFP KI model, single-cell RNA-seq data from Pgc-DsRed KI model, and single-cell RNA-seq data from Gif lineage tracing model are available from ArrayExpress under accession numbers ArrayExpress: E-MTAB-10373, E-MTAB-10371, and E-MTAB-11587 respectively.

The raw datasets/codes generated during time-course bulk RNA-seq and single-cell RNA-seq analysis are available from the following repository: https://github.com/scg-dgist/CELL-STEM-CELL-gastric-chief-cell. Any additional information required to reanalyze the data reported in this work paper is available from the lead contact upon request.

EXPERIMENTAL MODEL AND SUBJECT DETAILS

Animals

The *Troy-eGFP-IRES-CreERT2* (Troy-eGFP KI) mouse line (Stange et al., 2013) and the *Pgc-IRES-DTR-T2A-dsRed* (Pgc-DsRed KI) mouse line (Han et al., 2019) were used for time-course bulk RNA-seq and scRNA-seq, respectively. The *R26loxpTA-p57* mouse line (MGI ID:5492378) was obtained from The Jackson Laboratory. The *Anxa10-CreERT2* mouse line (Seidlitz et al., 2019) was used for stomach-specific overexpression or knockout of *p57*. To generate *Gif-rtTA-TetO-Cre-Gt(ROSA)26Sor*$^{tm1(CAG\text{-}tdTomato*,\text{-}EGFP*)Ees}$*/J (Gif-Cre-nTnG)* mice, the *Gif-rtTA* mice (Caldwell et al., 2021) were crossed against *TetO-Cre* mice then dual-positive mice were crossed with *Gt(ROSA)26Sor*$^{tm1(CAG\text{-}tdTomato*,\text{-}EGFP*)Ees}$*/J* (*Rosa26-nTnG*) mice to complete the allele of *Gif-Cre-nTnG*. They were used for lineage tracing and Gif lineage scRNA-seq. All mice were group-housed under specific pathogen-free conditions and had not previously undergone any procedures.

Generation of p57 conditional knock-out mice

To generate the conditional knock-out allele, we designed and synthesized a targeting construct containing two loxP sites flanking exon 2 of the *p57* gene with homology arms at both ends. We utilized two vectors of Cas9 nickase with gRNAs to induce homology-mediated recombination. After cloning the vectors, we transfected the constructs to mouse embryonic stem cells and picked the successful clones. Blastocyst injection was carried out at the IMBA/IMP Transgenic Facility.

Animal treatments

Anxa10-CreERT2; R26loxpTA-p57, Anxa10-CreERT2; p57$^{loxP/loxP}$, *Anxa10-CreERT2* control, and C57BL/6J mice were intraperitoneally injected with 5 mg per 20 g body weight of tamoxifen in corn oil to induce recombination and stomach damage at the same time. *GIF-Cre-RnTnG* mice were treated with doxycycline in drinking water at a concentration of 0.2 mg/ml for one week. Mice were left for two to three weeks for complete GFP-labeled chief cell maturation. Then parietal cell depletion was performed either by a single administration of DMP-777 (5.6 mg per 20 g body weight) or administration of L635 (7 mg per 20g body weight) once a day for 3 consecutive days, both by oral gavage. For the time-course analysis, parietal cell depletion was performed by DMP-777 administration by oral gavage (5.6 mg per 20 g body weight) in wild type mice using the time course described in the figures. We used both male and female mice (8-14 weeks of age) in all our experiments. The influence of sex was not considered in this study, as homeostatic tissue turnover exists in both sexes. All procedures were performed according to United Kingdom Home Office regulations and local animal welfare committee guidelines or according to the Austrian Animal Care and Use Committee.

Establishment and culture of gastric corpus organoids from mouse

Stomachs from C57BL/6J mice for generating Dox-inducible lines or from *Anxa10-CreERT2* or *Anxa10-CreERT2; p57*$^{loxP/loxP}$ for p57 knock-out organoid experiment were prepared by carefully separating the corpus from the forestomach and pylorus. Corpus tissue was then minced into small pieces in a petri dish using a scalpel or scissors. Corpus fragments were transferred into a Falcon tube with 10 ml Gentle Cell Dissociation Mix (STEMCELL Technologies) for 25 min incubation on a tube roller at RT. The tube containing the corpus fragments was then shaken vigorously before 5 min centrifugation at 300 g. The supernatant was discarded and the pellet including a small volume of remaining dissociation solution transferred to a small 3 cm petri dish using a cut P1000 pipette tip. A coverslip was gently placed on the tissue fragments and while observing under a microscope (EVOS), pressure was applied to the coverslip to dislocate the glands from the tissue fragments. Glands were transferred into a 15 ml Falcon tube by washing the coverslip and the petri dish two times with 5 ml PBS. The gland-containing solution was filtered through a 100 μm cell strainer before the glands were spun down for 5 min at 300 g. Depending on the size of the pellet, glands were resuspended in an appropriate volume of Matrigel and seeded as 20 μl droplets in prewarmed 48-well plates. Seeded glands were placed in the incubator for ~10 minutes before 250 μl pre-warmed complete medium (with 10 μM ROCK-Inhibitor) was added to each well. Final mouse stomach organoid culture medium (complete medium) contained 1x B27 (Invitrogen), N-acetylcysteine 1.25 mM (Sigma-Aldrich), EGF 50 ng/ml (Invitrogen), Noggin 100 ng/ml (Peprotech or Interpark), R-spondin1 conditioned medium 10%, Wnt3A conditioned medium 50% or recombinant Wnt-surrogate 0.5 nM (U-protein express; Janda et al., 2017), FGF-10 100 ng/ml (Peprotech), and gastrin 10 nM (Sigma-Aldrich) added to basal medium (Advanced Dulbecco's modified Eagle medium/F12 supplemented with

penicillin/streptomycin, HEPES, Glutamax) was used. For growth factor withdrawal experiments, specific growth factors were not added as indicated in the figures. For MEK inhibitor treatment, selumetinib (100 nM) or trametinib (1 nM) were added to the WNRG medium (-EGF-FGF).

Generation of Dox-inducible OE organoids

To induce p57 overexpression in the organoids, we utilized tetracycline (Tet)-ON technology to turn on gene expression upon doxycycline treatment (see Figure 4A). Using PiggyBac transposase, we introduced *CMV-rtTA-Hyg*^{R} and *CMV*$_{min}$*-TRE-p57-IRES-mCherry*, both flanked by transposon-specific inverted terminal repeat sequences (ITRs), into the genome, by electroporation of three DNA plasmids into the cells (Merenda et al., 2017). After recovery of the organoids for 7 days, we selected for clones containing *CMV-rtTA-Hyg*^{R} in hygromycin (100 μg/ml, InvivoGen)-containing medium for about 2 weeks. After selection, we picked colonies and grew each clone in a separate well. After duplication of the clones, we selected for *CMV*$_{min}$*-TRE-p57-IRES-mCherry* harboring organoids by mCherry expression upon treatment with doxycycline (1 μg/ml). p16-, p19^{ARF}-, p21-, and p27-expressing gastric organoids, as well as p57-expressing intestinal organoids, were generated using the same method.

METHOD DETAILS

Stomach preparation

Mice were euthanized by cervical dislocation or carbon dioxide inhalation and the stomach was harvested by dissection. The stomach was cut longitudinally following the greater curvature from the intestine to the esophagus and subsequently spread on a piece of cardboard, using needles to hold the tissue, before fixation in freshly prepared 4% PFA at 4 °C overnight (18 h) or in 10% neutral buffered formalin overnight at room temperature (RT) with shaking. After fixation, the stomach tissue was washed for 30 min three times with PBS at 4 °C with shaking.

Immunohistochemistry on paraffin sections

Paraffin-embedded sections (2 or 5 μm) were rehydrated, and the epitopes were exposed using sodium citrate (pH 6.0) at the VBC Histology Facility.

For immunohistochemistry with chromogens, sections were incubated in peroxidase blocking solution (3% H_2O_2) at RT for 10 min. After washing, sections were incubated in blocking solution (2% BSA, 5% goat serum, 0.3% Triton-X100 in PBS) for 1 h at RT. The following primary antibodies were used: mouse anti-H/K-ATPase (1:500; MBL, D031-3), rabbit anti-Ki67 (1:250; A. Menarini, MP-325-CRM1 or 1:200; Abcam, ab16667), rabbit anti-Gif (1:200; Sigma-Aldrich, HPA040774), rabbit anti-P57 (1:1000; Abcam, ab75974). The peroxidase-conjugated 2-step enhancer-polymer system (DCS, SuperVision 2 HRP Single Species) was used for detection.

For immunofluorescence, sections were blocked with blocking solution (2% normal donkey serum, 5% DMSO, 0.5% Triton X-100 in PBS) for 1 h at RT. The following primary antibodies were used: rabbit anti-p57 (1:250; Abcam, ab75974), rabbit anti-Gif (1:250; Sigma-Aldrich, HPA040774), rat anti-Ki67 (1:250; Invitrogen, eBioscience 14-5698-82 (Sol15A) or 1:200; Biolegend, 652402), goat anti-GFP (1:1000; Novus, NB100-1770ss), rabbit anti-Mist1 (1:1000; Cell Signaling, 14896S), mouse anti-p120 catenin (1:200; BD Biosciences, 610133) rat anti-CD44v9 (1:250; Cosmo Bio, CAC-LKG-M002), rabbit anti-pS6 240/244 (1:500; Cell Signaling, 5364), rat anti-Lamp1 (1:500; DSHB, 1D4B), FITC-conjugated-UEAI-Lectin (1:2000; Sigma, L9006). Alexa 647-conjugated GS-II Lectin (1:2,000; Invitrogen, L32451) and Alexa 488-conjugated GS-II Lectin (1:500; Invitrogen, L21415) were used for neck cell staining. The following secondary antibodies were used: donkey anti-rat IgG (H+L) Cross-adsorbed DyLight 650 (SA5-10029, Invitrogen), donkey anti-rabbit IgG H&L (Alexa Fluor® 488, Abcam ab150073), donkey anti-rabbit IgG (H+L) Highly Cross-Adsorbed Secondary Antibody, Alexa Fluor 546 (1:500, ThermoFisher, A10040), donkey anti-goat IgG (H+L) Highly Cross-Adsorbed Secondary Antibody, Alexa Fluor 488 (1:500, ThermoFisher, A-11055), donkey anti-rabbit IgG (H+L) Highly Cross-Adsorbed Secondary Antibody, donkey anti-mouse IgG (H+L) Highly Cross-Adsorbed Secondary Antibody, Alexa Fluor 790 (1:500, ThermoFisher, A11371).

Imaging

Brightfield imaging was scanned with Pannoramic FLASH 250II or Pannoramic FLASH 250III from the IMBA/IMP BioOptics facility. A 20/0.8 plan-apochromat objective was used for automated scanning. Regions of interest were cropped via CaseViewer software.

Fluorescent imaging was performed with a Leica SP8 DIVE microscope using confocal imaging units. 25 x water objective and 1.25-fold digital zoom were used in the LAS X software. The argon laser intensity was set to 30%. The X/Y resolution was set to 512X512 pixels. Resonant scanner option (High-speed 8 and 12 kHz) was selected and the line average was set to 48. Section images were processed and analyzed using ImageJ. Signal intensity was adjusted for analysis.

Cell dissociation for bulk RNA-seq and scRNA-seq

Stomachs were prepared by carefully separating the corpus from the forestomach and pylorus. Corpus tissue was divided into four pieces of similar size and incubated in 4 ml dissociation solution (45 U/ml Dispase II, Thermo Fisher Scientific; 0.6 mg/ml pancreatin, Sigma; 1x penicillin/streptomycin in DMEM high glucose, HEPES, no phenol red, Thermo Fisher Scientific) at 37 °C with shaking at 270 rpm. After dissociation, the solution becomes cloudy and corpus fragments appear more transparent. All subsequent steps were then performed on ice. To avoid cell loss due to cell adhesion to pipette or tube walls, all pipettes and tubes used for pipetting the cell suspension were pre-incubated with either DMEM or PBS containing FBS. To further disrupt the tissue, the cell suspension, including

CellPress
OPEN ACCESS

corpus pieces, was pipetted up and down several times using a 10 ml pipette. The cell suspension without remaining corpus pieces was then transferred to a 15 ml Falcon tube. The corpus pieces and the dissociation tube were then washed twice with 5 ml of 10% FBS in DMEM and the wash solution combined with the cell suspension for inactivation of the dissociation reaction. Cells were centrifuged at 300 g for 5 min and resuspended in 1% FBS in PBS. The cell suspension was filtered through a 100 μm cell strainer into a precoated 15 ml tube. The tube and filters were washed twice with 1 ml of 1% FBS in PBS and the cell suspension was then centrifuged a second time at 300 g for 5 min.

FACS sorting strategies for bulk RNA-seq and scRNA-seq

Stomach cell sorting was performed by the WT–MRC Stem Cell Institute Flow Cytometry Facility for bulk RNA-seq of Troy+ cells, by the WT Sanger Institute Flow Cytometry Facility for Pgc+ single-cell RNA seq, and by the IMP/IMBA Flow Cytometry Facility for Gif lineage single-cell RNA seq.

For Troy+ chief cell sorting, supernatant was removed and the cell pellet was resuspended in 100 μl antibody mix (1% FBS, 10 U / ml DNAse, 1 to 20 H/K-ATPase beta subunit (ATP4b) Alexa Fluor 555 rabbit antibody) for 1 h incubation on ice. Cells were washed with 3 ml 1% FBS in PBS and filtered once more if clumps could be observed. After 5 min centrifugation at 300 g, the cell pellet was resuspended in 1 ml 1% FBS and 10 U/ml DNAse in PBS containing and transferred to a MOFLO tube for sorting.

For scRNA-seq for Pgc+ cells, we utilized Pgc-IRES-DTR-T2A-dsRed knock-in mice as described previously (Han et al., 2019). After dissociation, the cell pellet was resuspended in 100 μl of antibody mix (1% FBS; 10 U/ml DNAse, 1:125 Alexa Fluor® 647-conjugated anti-mouse/human CD324 (E-Cadherin) antibody) and incubated for 1 h on ice. Cells were washed with 3 ml 1% FBS in PBS and filtered once more if clumps could be observed. After centrifugation at 300 g for 5 min the cell pellet was resuspended in 1 ml 1% FBS and 10 U/ml DNAse in PBS for sorting. BD INFLUX systems were used for sorting in the WT Sanger Institute Flow Cytometry Facility. After the viable cells were gated, the cells were subjected to the second gating to select only singlet cells. Then the third gating was used to select E-Cadherin+ epithelial cells by Alexa 647. The fourth gating was used to select Pgc+ cells by dsRed. Each well of the 384-well plate contained 2.3 μl of lysis buffer with RNAse inhibitor (Ambion) in 0.2% (v/v) Triton X-100. Preamplification was performed in a total volume of 27 μl containing 13.5 μl of HiFi Hotstart ReadyMix (2x; KAPA Biosystems) and 0.1 μM of IS PCR primer (Sigma-Aldrich). After 25 cycles of amplification, samples were cleaned with 80% (v/v) of Ampure beads (Beckman Coulter). Sequencing libraries were prepared using the Nextera XT DNA sample preparation kit (Illumina).

For scRNA-seq for Gif lineage cells, we utilized *Gif-rtTA; TetO-Cre; Rosa-nTnG* mice as described above. We administrated doxycycline by drinking water as described for animal treatments. Stomach samples from uninjured or DMP-777-treated mice at 2 dpi were collected separately and cells were dissociated. To sort GFP+ DsRed- cells only, we used WT stomach cells as a control to identify GFP+ DsRed+ cell windows in the FACS plot. A stepwise gating strategy is shown in Figure S3E. The cells were sorted in 384-well plates, called cell capture plates, that were ordered from Single Cell Discoveries, a single-cell sequencing service provider based in the Netherlands. Each well of a cell capture plate contains a small 50 nl droplet of barcoded primers and 10 μl of mineral oil (Sigma M8410). After sorting, plates were immediately spun and frozen at -80 °C until they were shipped in dry ice to Single Cell Discoveries.

RNA purification for bulk RNA-seq

For RNA purification, cells were sorted directly into 300 μl lysis buffer (Arcturus Pico Pure RNA Isolation Kit). Cell lysate was snap-frozen on dry ice and subsequently stored at -80 °C. RNA purification was performed using the Arcturus Pico Pure RNA Isolation Kit (Life Technologies) following the manufacturer's instructions.

Library preparation and bulk RNA-seq of Troy$^+$ chief cells

For single-end mRNA-sequencing, 17 total RNA samples were extracted from Troy$^+$-eGFP gastric corpus cells in homeostatic and injury conditions. The integrity of total RNA was confirmed using an Agilent BioAnalyzer. RNA integrity number (RIN) range was 6.6-9.3. cDNA libraries were generated from total RNA according to QuantSeq 3' mRNA-Seq FWD library preparation protocols. The library templates were amplified in 14-18 PCR cycles, and concurrently barcoded. Purification was performed with 27 μl PB, and the resulting end-point PCR products were analyzed on a bioanalyzer using a DNA HS chip. 6-10 fmol of each library (calculated from the 150-1500 nt region) were pooled to yield an equimolar lane mix. Each lane contained libraries from 12 or 13 samples. The lane mixes were separately subjected to single end 100 nt sequencing using an Illumina HiSeq 2500 system.

Generation of single-cell RNA-seq data

Library preparation for the Pgc+ cells was performed by the Single-Cell Genomics Core Facility of the WT Sanger Institute. Briefly, mRNAs isolated from single cells from each condition were amplified using the SMARTSeq2 protocol (Picelli et al., 2014). Multiplexed sequencing libraries were prepared from amplified cDNA using Nextera XT (Illumina) and sequenced on a HiSeq 2500 running in rapid mode.

Library preparation and sequencing of the Gif lineage cells were performed by Single Cell Discoveries. Single-cell RNA sequencing was performed according to an adapted version of the SORT-seq protocol (Muraro et al., 2016) with primers described in another paper (van den Brink et al., 2017). Cells were heat-lysed at 65 °C followed by cDNA synthesis. After second-strand cDNA synthesis, all the barcoded material from one plate was pooled into one library and amplified using in vitro transcription (IVT). Following amplification, library preparation was done following the CEL-Seq2 protocol (Hashimshony et al., 2016). To prepare a cDNA library for

sequencing, TruSeq small RNA primers (Illumina) were used. The DNA library was paired-end sequenced on an Illumina Nextseq™ 500, high output, with a 1x75 bp Illumina kit (read 1: 26 cycles, index read: 6 cycles, read 2: 60 cycles).

Immunostaining of p57-OE organoids

We performed whole-mount staining of gastric organoids as previously described (Mahe et al., 2013). Briefly, we seeded Dox-inducible p57-OE organoids on an 8-well chambered coverslip (Ibidi, 80826) and cultured them in complete medium with or without Dox. On the day of fixation, we removed the culture medium and washed the wells with PBS at RT. After removing PBS, cells were incubated in 200 µl of 4% PFA (at RT) for 30 mins. After washing with PBS three times, we proceeded with the blocking and staining procedure. The following antibodies were used for co-staining: rabbit anti-p57 (1:250; Abcam, ab75974), rat anti-Ki67 (1:250; Invitrogen, eBioscience 14-5698-82 (Sol15A)), donkey anti-rabbit IgG H&L (Alexa Fluor® 488, Abcam ab150073), and donkey anti-rat IgG (H+L) cross-adsorbed DyLight 650 (SA5-10029, Invitrogen).

Quantitative real-time PCR

Dox-inducible p57 OE organoids grown with or without Dox for 14 days were collected in lysis buffer of Qiagen RNeasy Micro kit. RNA was extracted according to the manufacturer's instructions. RNA was aliquoted to the volume of one-time use and stored at -80 °C. cDNA was synthesized with Oligo(dT)$_{18-22}$ primer by SuperScript III reverse transcriptase. Using GoTaq qPCR master mix, we performed qRT-PCR with 2 to 3 technical replicates for each marker and reference gene (Table S3). Marker genes of chief cells were selected from a list of DEGs from murine chief cells in a scRNA-seq data recently published (Busslinger et al., 2021). For the calculation, the common base method (Ganger et al., 2017) was used.

Transmission electron microscopy

Organoid samples were fixed using a mixture of 2% glutaraldehyde and 2% paraformaldehyde in 0.1 M sodium phosphate buffer, pH 7.2, for 2 h at RT and then overnight at 4 °C. Organoids were then rinsed with the same buffer, post-fixed in 2% osmium tetroxide 0.1 M sodium phosphate buffer, pH 7.2, on ice for 40 min, dehydrated in a graded series of acetone on ice and embedded in Agar 100 resin. 70 nm sections were cut and post-stained with 2% uranyl acetate and Reynolds' lead citrate. Sections were examined with an FEI Morgagni 268D (FEI, Eindhoven, The Netherlands) operated at 80 kV. Images were acquired using an 11-megapixel Morada CCD camera (Olympus-SIS).

QUANTIFICATION AND STATISTICAL ANALYSIS

Statistics

To quantify the injury-responsive cells, we counted 5-10 glands in the corpus zones for the 3 d HDT condition or all glands for the 14 d HDT condition except for the lesser curvature (which shows a different staining pattern from the center of the corpus) from each mouse stomach tissue section. In addition, the number of glands containing positive cells and the number of total glands were counted in one entire section for each condition to quantifiy the proportion of responsive glands. Corpus sections from 2-4 mice per condition were analyzed. The average values were compared by Kruskal–Wallis test or one-way ANOVA depending on the result of the normality test ($P<0.05$ was considered statistically significant). For quantification of mitochondria and granules in EM, 20 cells from 2 organoids were analyzed. The average values were then compared by Welch's test ($P<0.05$ was considered statistically significant).

Time-course RNA-seq data analysis

To check the quality of sequenced reads of 100 nucleotides from obtained mRNA-seq results, reads were imported into FastQC (Andrews, 2010). Based on the FastQC results, 7 nucleotides with low base signal quality at the 3' end and, if any, poly A-sequences were trimmed from the raw sequences. Additionally, random primer sequences (~12 nucleotides) at the 5' end were trimmed according to the guidance from the QuantSeq FWD manual. The trimmed reads were aligned to the mouse genome (GRCm38 from Ensemble) using STAR (Dobin et al., 2013) with up to 10 mismatches and 10 multiple alignments allowed. The aligned reads were indexed and sorted by SAMtools (Li et al., 2009). HTseq (Anders et al., 2015) was used to assemble the aligned reads to transcripts and quantify the read counts.

To analyze temporal expression patterns of genes across experimental time points, we used the TCseq (v1.10.0) R package (Wu and Gu, 2020). Raw read counts were normalized by trimmed mean of M-values and the time-course table of $\log_2$ fold changes (log2FC) of genes across all time points compared to the untreated control was obtained by using the timecourseTable function with the option of value= FC. Differentially expressed genes (DEGs) between each time point after DMP-777 treatment and the untreated control were identified using the generalized lineaer model of edgeR with adjusted $P < 0.01$ and absolute value of $\log_2 FC > 1$, implemented in the TCseq package. Genes with no significant changes were filtered out. The temporal patterns of log2FC were transformed to z-scores and divided into 6 clusters using the timeclust function with standardize=TRUE, k=6 and algo=cm (fuzzy c-means clustering). The number of clusters was chosen by calculating the Calinski-Harabasz index using the calinhara function of the fpc (v2.2-8) R package (Hennig, 2020) with the random seed of 6.

Functional enrichment analysis of DEGs at 1 dpi was performed using the topGO (v2.36.0) R package (Alexa and Rahnenfuhrer, 2019) with Gene Ontology Biological Process (GOBP) terms defined by the org.Mm.eg.db (v3.8.2) annotation data package (Carlson,

2019). Using the curated gene sets, we also performed gene set enrichment analysis (GSEA) on log2FC at 1 dpi using the GSEA software (v4.2.1) with default options except for max size=700. To perform GSEA, genes that were not expressed in any sample were excluded. The raw read counts were normalized by estimated size factors, which were calculated using estimateSizeFactors function of the DESeq2 R package (v1.26.0) (Love et al., 2014). The "QSC_UP_SIGNATURE" and "SPEM_UP_SIGNATURE" gene sets were obtained from REF1 and REF2, respectively. The "REACTOME_CELL_CYCLE" gene set was built by finding mouse orthologous genes of the "REACTOME_CELL_CYCLE" MSigDB gene set using the getLDS function of the biomaRt (v2.46.0) R package (Durinck et al., 2005; Liberzon et al., 2011). To build a gene signature for ISCs, we downloaded a dataset (GSE33949) from the Gene Expression Omnibus (GEO) database. The raw microarray gene expression data were preprocessed using the rma function of the oligo (v1.54.1) R package (Carvalho and Irizarry, 2010). Linear models were fitted to the normalized expression data using the lmFit function of the limma (v3.46.0) R package (Ritchie et al., 2015) and differential expression of each probe was statistically tested using the eBayes function of the same package with the option of trend=TRUE. DEGs were identified with adjusted $P < 0.05$ and absolute value of $\log_2 FC > 1$. The "ISC_UP_SIGNATURE" gene set was a set of upregulated genes in the $Lgr5^{hi}$ population compared to the $Lgr5^{lo}$ population.

To build gene signatures for gastric chief cells, we used two datasets (GSE133205 and GSE157694) from the GEO database and our scRNA-seq data. For GSE133205, we used the same method and parameters as for processing GSE33949. Genes that were more highly expressed in chief cells than in isthmus and parietal cells were selected. For GSE157694, we processed scRNA-seq data following our Pgc^{+} scRNA-seq data processing pipeline. Low-quality cells were removed with the number of detected genes lower than 1500 and reads assigned to ERCC higher than 20%. Highly variable genes (HVGs) were identified using the same method as above but the top 1000 variable genes were used. The batch effects for different plates were removed using the same method as scRNA-seq data analysis. All cells were visualized and clustered using top 15 PCs with default parameters. Then, a cluster representing chief cells was identified based on canonical marker genes and chief cell marker genes were identified using the FindMarkers function of Seurat R package satisfying average log2FC higher than 1 and adjusted *P* less than 0.05. For our Pgc+ scRNA-seq data, chief cell marker genes were identified from normal Pgc+ cells using the FindMarkers function satisfying average log2FC higher than 1.5 and adjusted *P* less than 0.01. The curated gene lists used in the GSEA analysis are provided in Table S2.

scRNA-seq data preprocessing

For Pgc+ scRNA-seq data, raw reads of Pgc+ gastric corpus cells during homeostasis and 1 dpi after DMP-777 treatment (dataset1) were mapped to the mouse reference genome (GRCm38) using STAR (v2.5.2b) with default options and the GTF file of GRCm38 provided by Ensembl (release 90). We also used 743 Pgc+ gastric corpus cells in homeostasis from our public scRNA-seq dataset (dataset2). A gene-by-cell count matrix was generated by quantifying uniquely mapped reads using htseq-count (v0.7.2). We filtered out poor quality cells with read counts less than 300,000, the number of genes less than 4,000 and reads assigned to mitochondrial genes higher than 10% using the CalculateQCMetrics function of the scater (v1.14.0) R package (McCarthy et al., 2017). For Gif lineage scRNA-seq data, using the zUMIs pipeline (Parekh et al., 2018), raw reads of cells were mapped to the mouse reference genome (GRCm38) and a gene-by-cell matrix was generated with the GTF file of GRCm38 provided by Ensembl (release 90) including GFP sequence. We filtered out poor quality cells with $\log_{10}$-scaled unique molecular identifiers (UMIs) less than 2.5 and reads assigned to ERCC higher than 20% using the same method as above. To remove cell-specific biases, cells were pooled based on their expression profiles using the quickCluster function of the scran (v1.14.6) R package (Lun et al., 2016) with default parameters and cell-specific size factors calculated using the computeSumFactors function of the same package. Raw read counts were normalized by dividing them with the estimated size factors and $\log_2$-transformed with the pseudocount of 1. To identify HVGs, an abundance-dependent trend to the variance of the log-normalized expression values was fitted using the trendVar function and the gene-specific variance was decomposed into its biological and technical components using the decomposeVar function. For Pgc+ scRNA-seq data, HVGs for dataset1 were defined as genes satisfying FDR less than 0.01 and biological variance larger than 0.5. For dataset2, genes with FDR less than 0.01 and biological variance larger than 0.2 were defined as HVGs. For Gif lineage scRNA-seq data, HVGs were defined as genes satisfying FDR less than 0.05 and biological variance larger than 0.05. To integrate scRNA-seq datasets from different plates, anchors between the datasets were identified using the FindIntegrationAnchors function of the Seurat (v3.1.4) R package (Butler et al., 2018; Stuart et al., 2019) with default parameters and the two datasets were integrated using the IntegrateData function. To reduce the cell cycle-dependent effect on cell-to-cell variability in gene expression, scores for S and G2M phase were regressed out using the ScaleData function with the option of vars.to.regress. HVGs were identified from the integrated scRNA- seq data using the same method as above. For Pgc+ scRNA-seq data and Gif lineage scRNA-seq data, the top 20 and 30 principal components (PCs) calculated from the integrated gene-by-cell matrix with HVGs were used for downstream analysis, respectively. A Shared Nearest Neighbor (SNN) graph was constructed using the FindNeighbors function with the default parameters except for k.param = 20 and 40, respectively. Based on the SNN graph, cells were clustered using the FindClusters function with resolution=1.5 and 0.8, respectively. Cells were visualized in the two-dimensional UMAP plot using the RunUMAP function. To compare the relative gene expression between clusters, the normalized data was scaled and average scaled expression per cluster was calculated in each condition.

scRNA-seq data analysis

To infer the direction of cellular differentiation based on the ratio of unspliced to spliced mRNAs, we performed RNA velocity analysis for each condition using the scVelo (v0.2.1) python package (Bergen et al., 2020). For each scRNA-seq dataset, the spliced and

unspliced expression matrices were generated using the run_smartseq2 function of the velocyto (v0.17.17) python package (La Manno et al., 2018) with the mouse reference masking GTF file (GRCm38) and the GTF file of GRCm38 provided by Ensembl (release 90). After filtering out low expressed genes, the matrices were normalized and natural log-transformed using the pp.filter_and_normalize function with default options except for n_top_genes=5000 for injury. Moments for velocity estimation were computed using the pp.moments function with the option of n_neighbors=20 and n_pcs=150 (for injury) or 30 (for control). RNA velocities were estimated by using the tl.velocity function with the option of mode=dynamical (for injury) or stochastic (for control). The velocity graphs were computed based on cosine similarities using the tl.velocity_graph function with default options. The RNA velocity results were visualized on the two-dimensional t-SNE plot using the Palantir (v0.2.6) python package (Setty et al., 2019). For the Palantir t-SNE plot, diffusion components (DCs) were computed using the run_diffusion_maps function with the first 100 PCs for injury and 200 PCs for control. Then, a k-nearest neighbor (kNN) graph (k=30) was constructed from the first 15 DCs for injury or 25 DCs for control. The coordinates for t-SNE plot were computed using the run_tsne function with the options of perplexity=100 (for injury) or 400 (for control). To quantify the connectivity between the 12 cell clusters, partition-based graph abstraction (PAGA) graph of each condition was generated using the tl.paga function of the scanpy (v1.5.1) python package (Wolf et al., 2018) with default options. The adjacency matrix was plotted using the pl.paga function with the options of threshold=0.35, random_state=1 and layout='fa' (ForceAtlas2) for both conditions.

To project Gif lineage cells onto Pgc+ stomach epithelial cells, for every Gif lineage cell, k-Nearest Neighbors (k-NNs) were obtained from Pgc+ stomach epithelial cells based on Pearson correlation coefficients of normalized expression data of HVGs between Gif lineage cells and Pgc+ stomach epithelial cells using the knn.index.dist function of the KernelKnn (v1.1.2) R package (Mouselimis, 2021). To obtain the projection, for every Gif lineage cell, two-dimensional coordinates of 10-NNs on the UMAP plot of Pgc+ epithelial cells were averaged.

For the Gif lineage cells, pseudotime and a differentiation trajectory were inferred using the slingshot (v1.4.0) R package (Street et al., 2018) based on the coordinates of the Gif lineage cells which were projected onto Pgc+ stomach epithelial cells. To obtain a differentiation trajectory, the starting cluster was set to 3 end ending clusters were not set. For each gene, gene expression was z-scored and plotted using a moving average along each of the trajectory with a window size of 100, which is 5% of the number of cells.

Cell Stem Cell, Volume *29*

Supplemental Information

p57^{Kip2} imposes the reserve stem cell state of gastric chief cells

Ji-Hyun Lee, Somi Kim, Seungmin Han, Jimin Min, Brianna Caldwell, Aileen-Diane Bamford, Andreia Sofia Batista Rocha, JinYoung Park, Sieun Lee, Szu-Hsien Sam Wu, Heetak Lee, Juergen Fink, Sandra Pilat-Carotta, Jihoon Kim, Manon Josserand, Réka Szep-Bakonyi, Yohan An, Young Seok Ju, Anna Philpott, Benjamin D. Simons, Daniel E. Stange, Eunyoung Choi, Bon-Kyoung Koo, and Jong Kyoung Kim

Figure S1

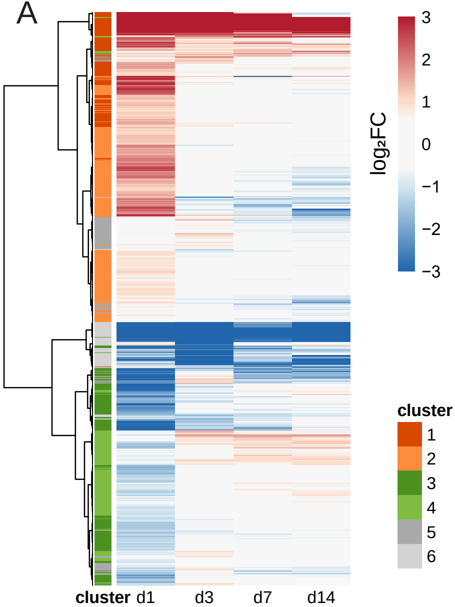

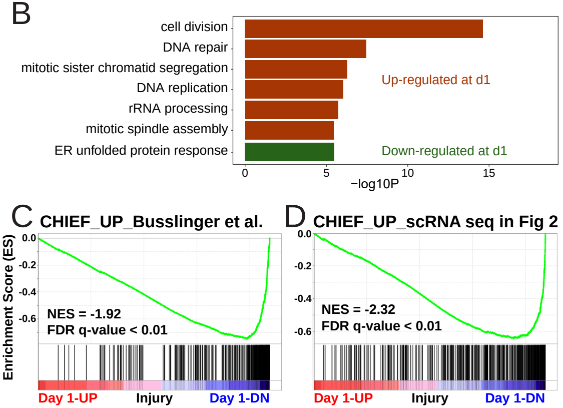

E

Source	Stange *et al.*, 2013	Leushacke *et al.*, 2017	Hata *et al.*, 2020	Busslinger *et al.*, 2021
Context	Troy+ chief cell / whole corpus	Lgr5high cell/ Lgr5- cell	Chief cell / parietal cell	Chief cell (Chia+ or Chia-) / other cells
p57 expression (log_2FC)	1.9	1.1	4.0	1.7 (Chia+); 1.5 (Chia-)

Figure S2

A

Cluster #	11	1	4	0	9	8	2	6	3	5	7	10	Total
	Chief	C/N-1	C/N-2	Neck	C/Pr-1	C/Pr-2	Pr-2	Pr-1	N/P	Pr/P-2	Pr/P-1	Pit	Total
Control	54	144	145	217	56	46	53	41	124	56	50	44	1030
Injury	11	31	7	2	39	54	110	81	32	76	68	42	553

B

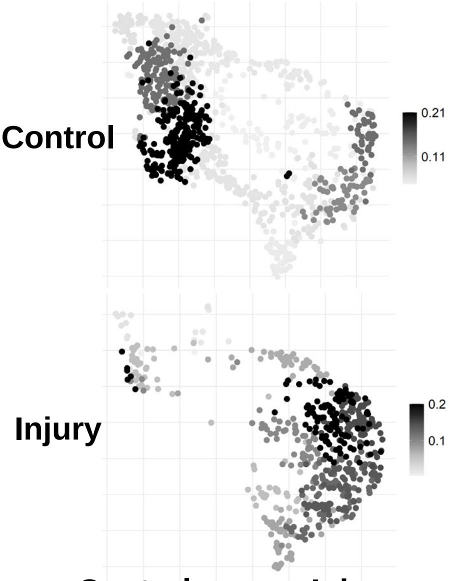

C

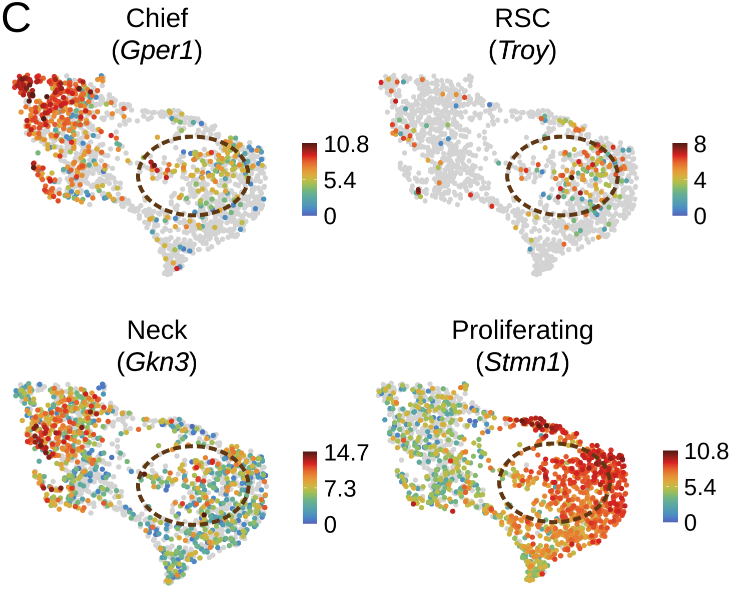

D

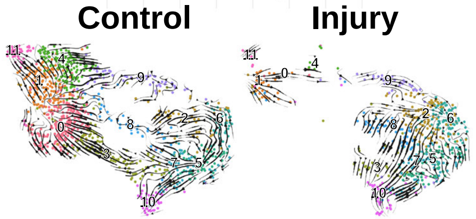

E

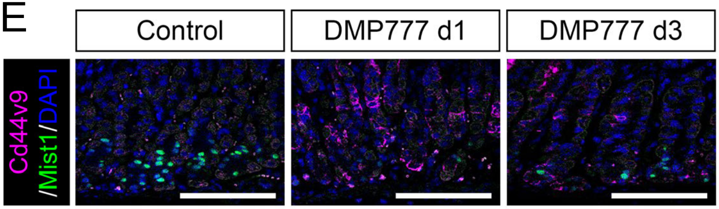

Figure S3

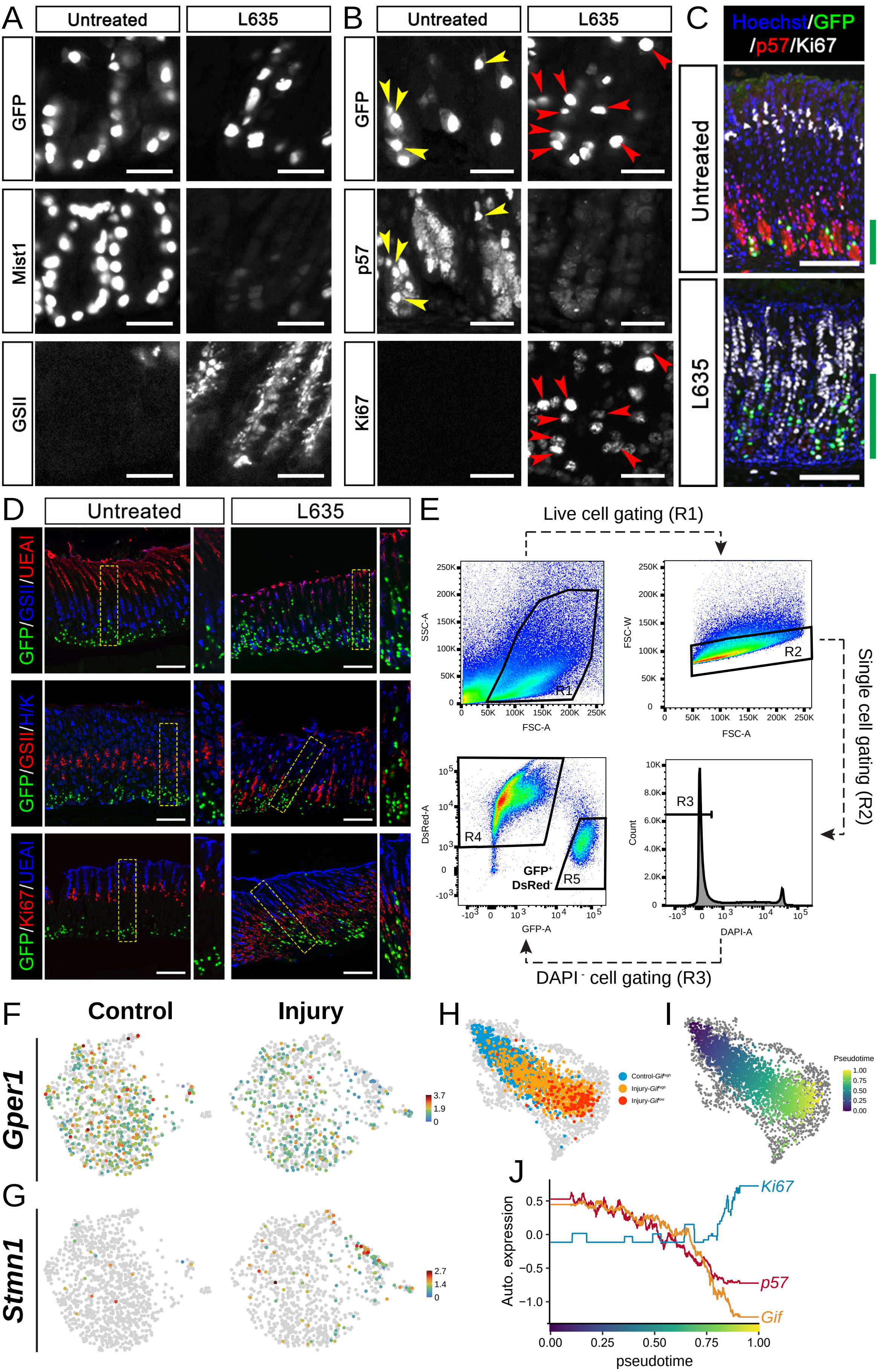

A
B
C
D
E
F
G
H
I
J
Untreated
L635
GFP
Mist1
GSII
p57
Ki67
Hoechst/GFP/p57/Ki67
GFP/GSII/UEAI
GFP/GSII/H/K
GFP/Ki67/UEAI
Live cell gating (R1)
Single cell gating (R2)
DAPI- cell gating (R3)
SSC-A
FSC-A
FSC-W
DsRed-A
GFP-A
Count
DAPI-A
R1
R2
R3
R4
R5
GFP+ DsRed-
Control
Injury
Gper1
Stmn1
Control-Gif high
Injury-Gif high
Injury-Gif low
Pseudotime
Auto. expression
pseudotime
Ki67
p57
Gif

Figure S4

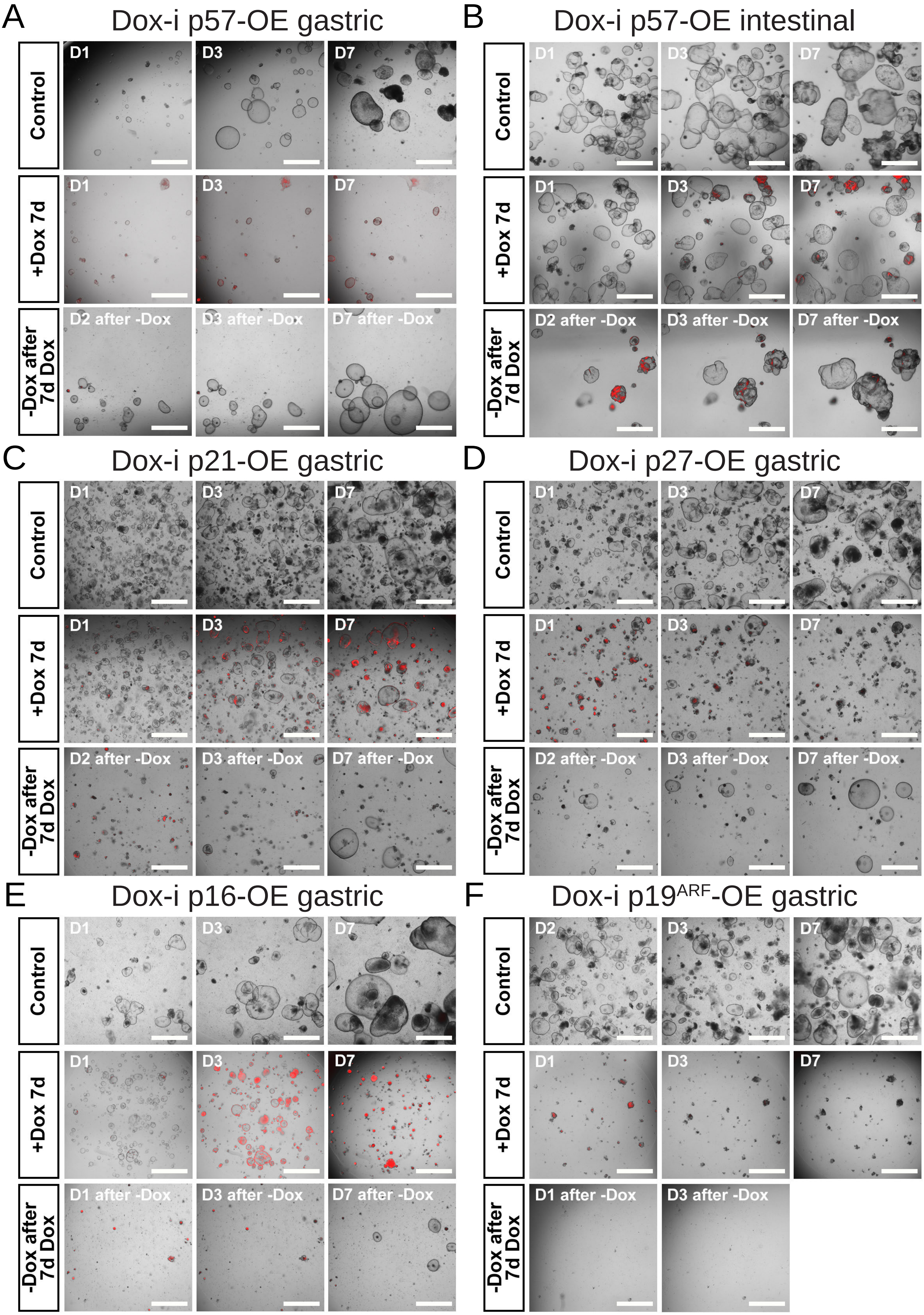
A
Dox-i p57-OE gastric
B
Dox-i p57-OE intestinal
Control
+Dox 7d
-Dox after 7d Dox
D1
D3
D7
D2 after -Dox
D3 after -Dox
D7 after -Dox
C
Dox-i p21-OE gastric
D
Dox-i p27-OE gastric
E
Dox-i p16-OE gastric
F
Dox-i p19ARF-OE gastric
D2
D1 after -Dox

Figure S5

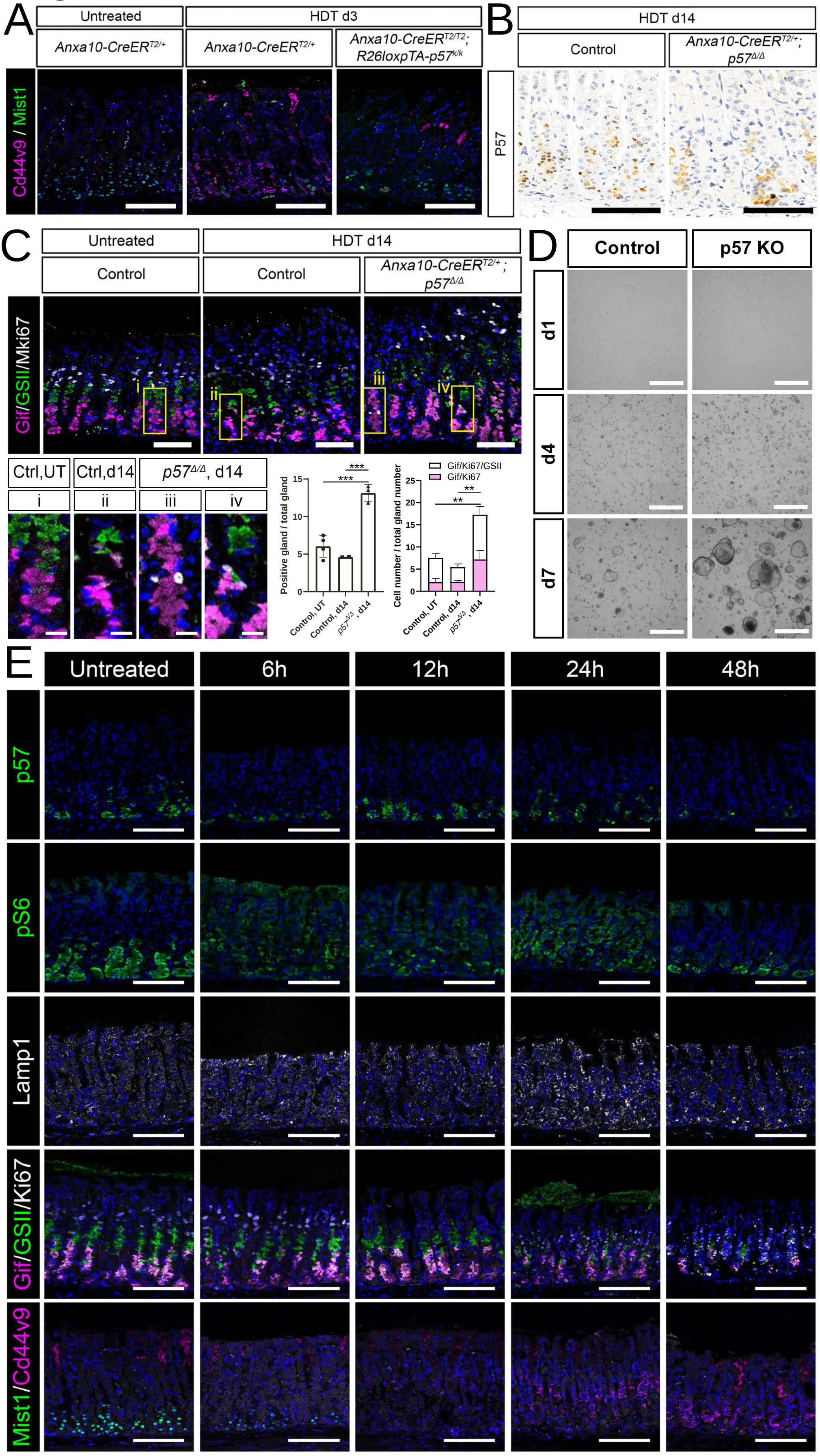

A
Untreated
HDT d3
Anxa10-CreERT2/+
Anxa10-CreERT2/+
Anxa10-CreERT2/T2; R26loxpTA-p57k/k
Cd44v9 / Mist1
B
HDT d14
Control
Anxa10-CreERT2/+; p57Δ/Δ
P57
C
Untreated
HDT d14
Control
Control
Anxa10-CreERT2/+; p57Δ/Δ
Gif/GSII/Mki67
i
ii
iii
iv
Ctrl,UT
Ctrl,d14
p57Δ/Δ, d14
Positive gland / total gland
Control, UT
Control, d14
p57Δ/Δ, d14
Gif/Ki67/GSII
Gif/Ki67
Cell number / total gland number
D
Control
p57 KO
d1
d4
d7
E
Untreated
6h
12h
24h
48h
p57
pS6
Lamp1
Gif/GSII/Ki67
Mist1/Cd44v9

Supplementary figure legends

Supplementary figure 1. Rapid transcriptome changes after injury shown in time-course bulk RNA-seq of Troy+ chief cells and enrichment of p57 expression in chief cells in homeostasis. (A) Heatmap of 1667 DEGs clustered by 6 patterns. 2-6 mice per time point were analyzed. Cluster 1 and 2 show peak expression at 1 dpi and gradual reduction during recovery. Cluster 3 and 4 show the biggest decrease in gene expression at 1 dpi. (B) Selected GO-terms enriched in upregulated (red) or downregulated (green) genes at 1 dpi (P-value < 10^{-5}). (C and D) GSEA of gene signatures associated with the chief cell signature from the scRNA-seq dataset from Busslinger et al., 2021 (C) and from the Pgc+ scRNA-seq dataset in Figure 2 (D). NES, Normalized Enrichment Score. (E) Enrichment of *p57* expression in chief cells in homeostasis from other datasets. Related to Figure 1, Tables S1 and S2.

Supplementary figure 2. Rapid switch of p57+ gastric chief cells to Ki67+ injury-responsive chief cells upon injury. (A) Cell numbers of each cluster in control and injury analyzed in Pgc+ scRNA-seq. (B) The proportion of the cell number of each cluster in control and injury samples. (C) UMAP plots of further markers for chief cells (*Gper1*), RSCs (*Troy*), neck cells (*Gkn3*), and proliferating cell markers (*Stmn1*). Brown dotted circles show injury-responsive chief cells. (D) RNA velocity inferred by scVelo projected on the UMAP plots in control and injury. (E) Double labelling of markers for SPEM (Cd44v9, magenta) and chief cells (Mist1, green) in control, at 1 dpi, and at 3 dpi. Nuclei were counterstained with DAPI (blue). Scale bars, 100 µm. Related to Figure 2.

Supplementary figure 3. Gif+ lineage tracing shows that Gif+ chief cells generate other cell types and acquire injury-responsive chief cell characteristics upon injury. (A and B) Single-channel images of Figure 3C and 3D, respectively. Scale bars, 20 µm. (C) Enlarged images of Fig 3D to show lineage tracing of Gif+ chief cells in control and injury. Green lines at the right show vertical expansion of the lineage tracing of Gif+ cells. Scale bars, 100 µm. (D) Examples of full gland lineage tracing of Gif+ chief cells (GFP). GSII, neck cell marker; UEAI, pit cell marker; H/K, H/K-ATPase, parietal cell marker. Scale bars, 100 µm. (E) Sorting strategy of Gif lineage cells (GFP^{+} $DsRed^{-}$) from *Gif-Cre-nTnG* mice. (F) UMAP plots of a further marker for chief cells (*Gper1*) in control (left) and injury (right). (G) UMAP plots of a further marker for proliferating cells (*Stmn1*) in control (left) and injury (right). (H) Projection of Gif^{high} cells in control and Gif^{high} and Gif^{low} cells in injury on the UMAP plot for Pgc+ scRNA-seq. (I) Pseudotime analysis of the Gif lineage cells in the projected UMAP of Pgc+ scRNA-seq data. (J) Gene expression trajectories along the

pseudotime trajectory. The represented expression values are log2-transformed normalized read counts followed by the z transform. The pseudotime is denoted in the bar on the x axis. The expression of *Ki67* gets increased while the expression of *p57* and *Gif* gets decreased along the pseudotime trajectory. Related to Figure 3.

Supplementary figure 4. Regrowth assay after expressing cell cycle inhibitors in gastric organoids and p57 in intestinal organoids by Dox-inducible system. (A and B) 7d of Dox treatment and Dox withdrawal experiments in Dox-i p57-OE gastric organoids (A) and in Dox-i p57-OE intestinal organoids (B). (C and D) 7d of Dox treatment and Dox withdrawal experiments in Dox-inducible CIP/KIP family CKI expression. (C) Dox-i p21-OE gastric organoids. (D) Dox-i p27-OE gastric organoids. (E) 7d of Dox treatment and Dox withdrawal experiments in Dox-inducible expression of INK4 family of CKI, p16 in gastric organoids. (F) 7d of Dox treatment and Dox withdrawal experiments in Dox-inducible expression of $p19^{ARF}$ in gastric organoids. Scale bars, 1 mm. Related to Figure 4.

Supplementary figure 5. Injury response is inhibited in p57 OE and prolonged in p57 KO *in vivo*. (A) Double staining of markers for SPEM (Cd44v9, magenta) and chief cells (Mist1, green) in the conditions as outlined above. Scale bars, 100 µm. (B) p57 staining in control and p57 knockout epithelium after 14 d of HDT treatment. Scale bars, 100 µm. (C) Triple staining with markers for chief cells (Gif, magenta), neck cells (GSII, green), and proliferating cells (Ki67, white) and quantification of injury responsive chief cells. 2-4 mice per condition were analyzed. Scale bars, 100 µm for the upper figure sets, 20 µm for the insets. Data in the graphs are represented as mean ± SD. $^{**}P<0.01$ and $^{***}P< 0.001$ calculated by one-way ANOVA. (D) Growth difference between control and p57 knockout organoids. Scale bars, 1 mm. (E) Labelling with several markers for the metabolic and molecular changes of chief cell transition upon injury. Scale bars, 100 µm. Related to Figure 5.
